# The Deep Subsurface Biosphere and its Substrates Along a One-Million-Year Ferruginous Lake Archive

**DOI:** 10.1007/s00248-025-02559-4

**Published:** 2025-06-03

**Authors:** Fátima Ruiz-Blas, André Friese, Alexander Bartholomäus, Cynthia Henny, James M. Russell, Jens Kallmeyer, Aurèle Vuillemin

**Affiliations:** 1 Section Geomicrobiology, GFZ Helmholtz Centre for Geosciences, Telegrafenberg, 14473 Potsdam, Germany; 2 Research Center for Limnology and Water Resources, National Research and Innovation Agency (BRIN), Republic of Indonesia, Cibinong, 16911 Jawa Barat Indonesia; 3 Department of Earth, Environmental, and Planetary Sciences, Brown University, 324 Brook Street, Providence, RI USA

**Keywords:** Deep biosphere, Pore water geochemistry, Volatile fatty acids, Organic matter remineralization, Bathyarchaeia, Ferruginous Lake Towuti

## Abstract

Lake Towuti, Indonesia, is an ancient stratified lake with ferruginous (iron-rich, sulfate-poor) anoxic bottom water conditions and a long depositional record affected by redox changes in the water column and sediments. As modern analogue of Earth’s early ferruginous oceans, it enables the study of an active microbial subsurface biosphere and its role in organic matter and iron mineralization. Combining 16S rRNA genes, cell counts, pore water geochemistry, and bulk sediment profiles from a 100-m-long core, we present the first comprehensive characterization of the deep subsurface biosphere along a one-million-year lacustrine archive. Electron acceptors in the pore water became depleted at shallow depths, resulting in a drastic decrease in cell densities in the fermentative zone, where Bathyarchaeia dominate the microbial community composition. Although alpha and beta diversity reflected initial depletion of substrates during burial, they also varied across successive lithologies, indicating that sediment composition subsequent to deposition also affects diversity. The upper sediments (0–20 mblf) sheltered a dense and diverse microbial community involved in organic matter remineralization, actively producing and converting volatile fatty acids into carbon dioxide and methane. Deeper sediments (20–70 mblf) contained low-diversity microbial communities adapted to nutrient scarcity. In contrast, deepest lacustrine sediments (70–100 mblf) contained an increased microbial diversity reflecting greater availability of organic matter of terrestrial origin. Despite Bathyarchaeia being prime constituents of the deep subsurface biosphere, increased diversity in 16S rRNA gene composition was observed in discrete sediment layers (tephra, diatom ooze, peat). This demonstrated that depositional conditions remained traceable, while stratified microbial communities drove reductive diagenesis.

**Supplementary Information:**

The online version contains supplementary material available at 10.1007/s00248-025-02559-4.

## Introduction

The deep biosphere, encompassing mainly microbial life whose metabolic limits require further exploration in subsurface environments, is a frontier in understanding global biogeochemical cycles [1–3]. Previous studies have mostly focused on the marine subseafloor, oceanic crust and hydrothermal vents, which are environments characterized by substrate limitation [4–6], and yet they were found to host diverse microbial communities that play crucial roles in Earth’s nutrient turnover [7, 8] and conversion of buried organic matter (OM) into methane and carbon dioxide (CO_2_). However, the lacustrine deep biosphere remains poorly characterized in terms of abundance, diversity, and metabolic traits [9–12], and its role in nutrient cycling and redox processes during long-term burial remains elusive.

Lakes are dynamic ecosystems, and microbial populations in the benthos and subsurface tend to respond to climate- or tectonically driven depositional changes that directly determine the nature of sedimentary substrates which, in turn, affect the distribution and diversity of the subsurface biosphere [9–11]. Over time, the gradual depletion in bioavailable substrates and decreasing cell densities result in changes in microbial community composition along a vertical gradient [13] towards a deep biosphere assembly [14]. By driving early diagenetic processes in the sediment, shallow and deep microbial populations are expected to correlate with variations in pore water geochemistry and induce authigenic mineral formation in discrete layers [15].

**Fig. 1:**
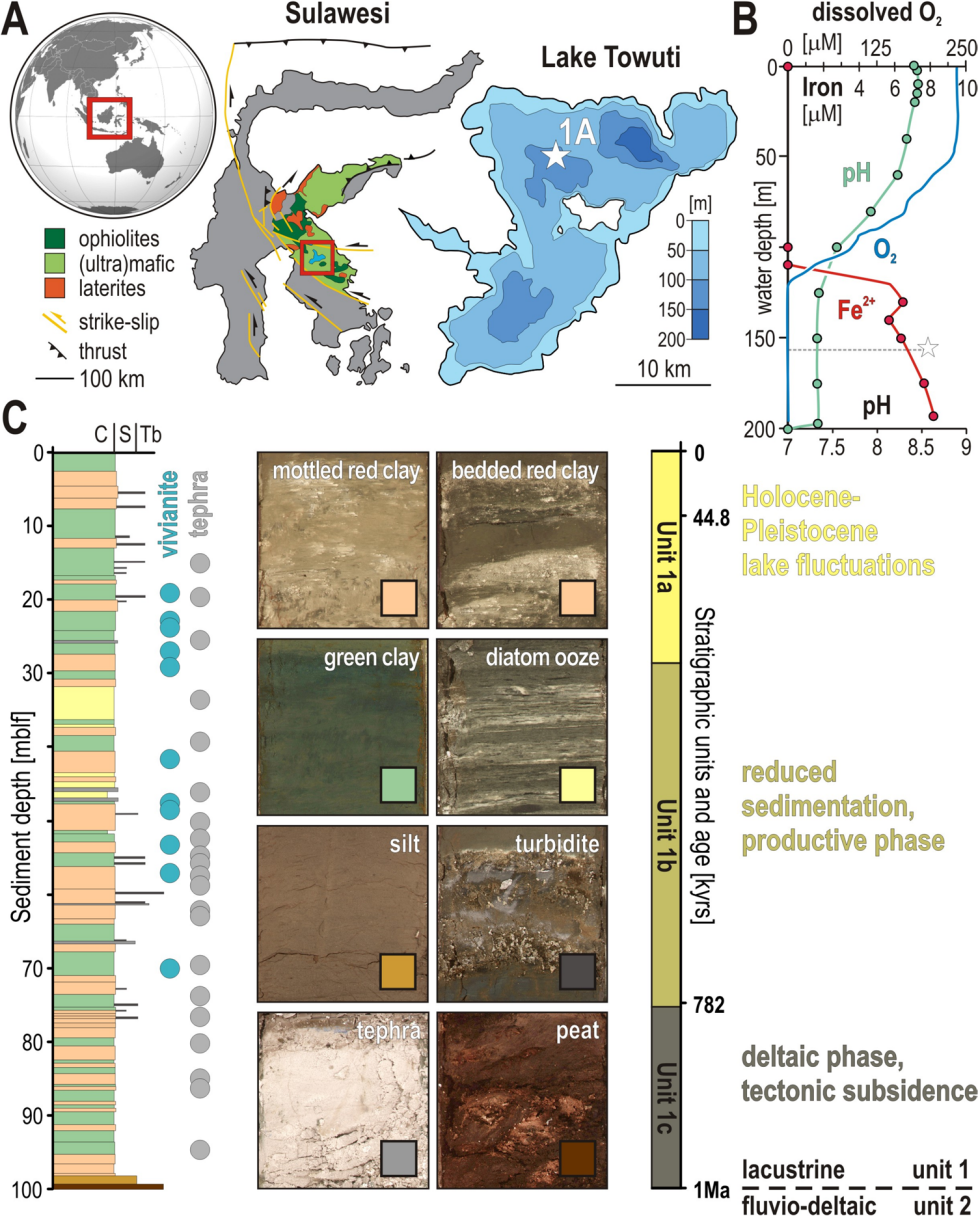
Lake Towuti site description, geological context and stratigraphic record. **A** World map with location (square) of Sulawesi, geology in the catchment of the Malili Lake System, and bathymetry of Lake Towuti. The star marks the position of the drill site 1A corresponding to 156 m water depth. **B** Lake Towuti’s water column profiles for oxygen and iron concentrations, and pH. **C** Stratigraphic log of the 100-m-long sediment core TDP-1A detailing the different lithologies and the three stratigraphic units over a million-year sedimentation. The figure is modified from [25, 28]

Due to its unique geochemical conditions and long-term depositional history [16], Lake Towuti, Sulawesi, Indonesia (Fig. 1A), provides an exceptional environmental setting for deep biosphere studies, with strong redox gradients that shape microbial community distribution in the water column as well as in the sediment [17]. Weathering of Lake Towuti’s ultramafic tropical catchment delivers only minimal sulfate to the lake [18] but causes high fluxes of iron oxides to the water column that scavenge bioavailable phosphorus therein [19], thereby maintaining ultra-oligotrophic conditions. The presence of a permanent oxycline (Fig. 1B) results in anoxic ferruginous conditions in bottom waters [20], making modern Lake Towuti a ferruginous analogue for microbial life under depositional conditions similar to those of the Proterozoic oceans [21]. The sediment harbors an active subsurface biosphere [22, 23] known to exhibit adaptation to sulfate-poor, iron-rich conditions [24, 25] that can be expected to persist over long-term burial. Thus, modern ferruginous Lake Towuti and its sediment provides a unique opportunity to explore how ferruginous conditions influence the microbial subsurface biosphere at the time of deposition and constrain its role in biogeochemical processes over a long depositional record.

Here, we hypothesize that fluctuations in Lake Towuti’s aquatic ecosystem modulate both sedimentation and redox conditions at the sediment–water interface (SWI), which become traceable as selective assemblages constituting microbiomes residing in the sediment [10, 11], potentially recording one million years (1 Ma) of depositional history [26, 27]. By combining high-throughput sequencing of 16S rRNA genes with total cell counts, pore water geochemistry, and bulk sediment profiles, we present a comprehensive characterization of the lacustrine deep subsurface biosphere, using Lake Towuti as case study. We detail how sediment microbial communities reacted on changes in depositional conditions at the time of burial, trace post-depositional selection of microbial communities, and address their role in early diagenesis, such as organic matter (OM) remineralization and iron mineralization. Our study provides insights into geomicrobiological interactions in a modern ferruginous environment and its evolution over 1 Ma to better constrain interpretations of lacustrine records, sediment biogeochemical cycles, and early diagenesis of iron-rich deposits [15, 28].

## Material and Methods

### Lake Towuti as a Ferruginous Case Study

Lake Towuti, situated in Central Sulawesi, Indonesia (2°45'S, 121°30'E), is part of the Malili Lake System, which lies within a tropical rainforest of high biodiversity and exhibits unique climatic and limnological properties [17, 29]. With a surface area of ca. 561 km^2^ and a maximum water depth of 203 m, Lake Towuti is one of the largest tectonic lakes in the world.

Since the Mid-Pleistocene, Lake Towuti has undergone several climate- and tectonic-driven shifts in trophic state and redox conditions that were recorded as an alternation of red siderite-rich and green vivianite-bearing clay layers [28, 30–33]. Lake Towuti's lacustrine record (Unit 1) has been divided into three stratigraphic units (Unit 1a to 1c), which correspond to different sedimentation regimes resulting from significant geological and environmental changes [17, 29], underlain by a riverine sediment sequence (Unit 2). The upper unit (Unit 1a) is characterized by lake level fluctuations related to the last glacial-interglacial cycle. The middle unit (Unit 1b) represents a phase of reduced sedimentation with high nutrient turnover that enhanced diatom productivity [34]. The basal unit (Unit 1c) reflects early tectonic subsidence of the lake basin with shallow(er) lacustrine sedimentation, which is underlain by a fluvial sequence (Unit 2) recording pre-lacustrine, riverine, deltaic and terrestrial conditions. Each of the three upper subunits exhibits characteristic lithologies, including red and green clay, diatom ooze, tephra, turbidite, ending with a peat layer at the base of Unit 1c (Fig. 1C). Because microbial influence on pore water geochemistry results in sequential precipitation of authigenic phases (i.e., magnetite, siderite, millerite, vivianite) during early burial [15], their alternation and relative abundances along the sedimentary sequence inform on bottom water redox conditions [20, 22] and substrates at the time of deposition [28]. Thus, the stratigraphic distribution of lithotypes along the sediment sequence should enable linking a long and complex succession of environmental changes [16, 17] to biogeochemical cycling by an entombed, yet potentially active and persistent subsurface biosphere.

### Water Column Casts, Drilling Operations, and Geomicrobiology Sampling

On-site measurements of oxygen concentration in the water column were taken using a submersible conductivity-temperature-depth probe (Sea-Bird SBE-19, Sea-Bird Electronics). Water samples were collected in 5 L Niskin bottles (General Oceanics, Miami, FL, USA). Water column pH, and Fe^2+^ concentrations were measured with a portable pH meter, and spectrophotometer, as previously published [20].

In the frame of the International Continental scientific Drilling Program (ICDP) Towuti Drilling Project (TDP), the sampling site TDP-1 (Fig. 1A) was chosen as the main drill site as its location at 156 m water depth was foreseen to have recorded depth fluctuations in the oxycline and associated redox conditions at the SWI. In May 2015, the ICDP Deep Lakes Drilling System (DLDS) retrieved cores from the 113-m-deep hole TDP-1A, covering about 1 Ma of sedimentation history [16]. Drilling operations involved the use of a hydraulic piston corer (HPC), which allowed for the retrieval of high-quality, undisturbed cores [17]. We also added a contamination tracer to the drilling fluid [35] to ensure that only those parts of the core that were not infiltrated by the drilling fluid were used for subsequent geochemical and biological analyses. In the field, sediment samples were collected as whole round cores (WRC) cut from the liners, which were immediately capped and directly transferred into an anaerobic chamber on site for further processing and aliquoting. Sediment samples were taken for pore water geochemistry, microbial cell counts, sulfate reduction rates (SRR), and DNA extraction, as previously described [22]. Sediment for genomic analysis was transferred into aluminum foil bags flushed with N_2_, heat-sealed and stored at room temperature until extraction in the home lab.

### Bulk Sediment and Pore Water Geochemistry

Total nitrogen (TN) and total organic carbon (TOC) concentrations were measured on freeze-dried bulk sediment with a Vario EL III CHNS elemental analyzer (Elementar GmbH, Hanau, Germany). Pore water Ca^2+^, Mg^2+^, Cl^−^, SO_4_^2−^, and NH_4_^+^ concentrations were measured using suppressed (anions) and non-suppressed (cations) ion chromatography [22, 28]. Pore water PO_4_^3−^ and Fe^2+^ concentrations were measured using spectrophotometry [28]. Sediment samples for pore water CH_4_ concentrations were taken on board the drilling platform immediately after retrieval of the core. Samples with a volume of 3 cm^3^ were taken with tip-cut syringes from the freshly cut ends of core sections and placed in 10 ml glass crimp vials pre-filled with saturated NaCl solution. Vials were completely filled without any headspace. This way of sample fixation also allows measuring CO_2_, which is not possible when fixing the sample with NaOH or leaving an air-containing headspace. The samples were measured via gas chromatography in the home lab, after introducing a 2 ml He-headspace into the vial and at least 24 h of equilibration [36]. Pore water concentrations of volatile fatty acids (VFAs) (i.e., lactate, formate, acetate, propionate, and butyrate) were measured using 2D-ion chromatography-mass spectrometry [37]. SRR were determined by incubating sediment with radioactive ^35^SO_4_^2−^. The biologically produced reduced sulfur species were separated using cold chromium distillation and quantified via scintillation counting [36, 38].

Total cell counts were determined on sediment samples fixed in a 2% formalin solution. A fixed amount of sediment slurry was processed for cell extraction, stained with SYBR Green I, and cells counted via epifluorescence microscopy, as previously published [22, 38]. Contrarily to DAPI and acridine orange, SYBR Green I only stains double-stranded DNA, which we use as a marker for intact cells [38]. All methodological procedures have been previously published [22, 28, 36].

### Aliquoting for DNA Extraction

The sealed aluminum foil bags containing WRC samples were stored at room temperature (25 °C) which was comparable to that of lake bottom waters (28 °C). The sealed bags were opened inside an anaerobic chamber at GFZ Potsdam. Samples showing any sign of oxidation were discarded and not processed any further. Three subsamples of ca. 20 g each were taken from every WRC, using sterile cut-off syringes. These mini-cores were placed in aluminum foil bags, heat-sealed inside the anaerobic chamber to avoid introduction of oxygen and stored in the −80 ºC freezer until DNA extraction.

For the upper 50 cm below lake floor (mblf), total DNA was extracted from 1 g of sediment, using the GeneMATRIX Environmental DNA & RNA Purification Kit (EURx®, Gdánsk, Poland), and processed undiluted for PCR amplification of partial 16S rRNA genes, as previously published [25]. For sediments between 1 and 5 mblf, total DNA was extracted from ca. 2 g of sediment using the DNeasy PowerSoil, and processed undiluted for PCR amplification. Below 5 mblf down to 113 mblf from ca. 10 g of sediment using the DNeasy PowerMax Soil Kit (Qiagen, Hilden, Germany), following the manufacturer’s instructions. The final DNA elutions were concentrated from 5 mL into 200 μL, using Amicon Ultra Centrifugal Filters of 30 kDa (Merck KGaA, Darmstadt, Germany) and diluted 10-fold for PCR amplification. For multiple samples (at 13.5, 45.4, 48.5, 68.2, 76.7, 81.2, 87.2, 91.2, 99.3, 103.3, 110.7, and 113.0 mblf), we had to perform a second round of DNA extraction from 20 g of sediment (2 reaction kits, final elution in 10 mL), and pooled their final concentrated extracts to ensure sufficient DNA yield for downstream sequencing of 16S rRNA gene amplicons. These extracts were diluted 100-fold for PCR amplification.

### PCR Amplification and 16S rRNA Gene Libraries

PCR amplification was performed using unique combinations of the barcoded universal primer pair 515F (5′-GTG TGY CAG CMG CCG CGG TAA-3′) with 806R (5′-CCG GAC TAC NVG GGT WTC TAA T-3′) as previously published [39]. The PCR products were cleaned using the HighPrep PCR Clean-up system (MagBio Genomics Inc., Gaithersburg MD, USA) and pooled, adding 20 ng of each PCR cleaned product per sample, and sent out to two commercial companies for high-throughput sequencing.

For DNA extracts from the gravity core, partial 16S rRNA gene amplicons (2 × 250 bps) were sequenced at Novogene (Novogene GmbH, Munich, Germany) on an Illumina NovaSeq platform (Illumina, San Diego, California, USA). For DNA extracts from core TPD-1A as well as 3 samples from the gravity core (at 0.05, 0.17 and 0.38 mblf), partial 16S rRNA gene amplicons (2 × 300 bps) were sequenced at Eurofins (Genomics Europe Shared Services GmbH, Ebersberg, Germany) on an Illumina HiSeq machine with MiSeq V3 chemistry. We could thereby compare both sequencing outputs and process them together. Two mock communities and negative controls for sample extraction and PCR amplification were systematically processed in parallel and sequenced (Supplementary Data S1).

Sequencing of 16S rRNA gene libraries performed on biological replicates in 2015, 2019, and 2022 yielded identical results [25, 39], ensuring that long-term storage at room temperature did not alter the microbial composition of sediment samples.

### Bioinformatics Treatment and Statistical Analyses

Read demultiplexing was performed with Cutadapt v. 3.5 [40]. Amplicon sequence variants (ASVs) were generated using the DADA2 package v. 1.20 [41] with R v. 4.1, applying the pooled approach with the following parameters: truncLen = c(220,180), maxN = 0, rm.phix = TRUE, minLen = 160. Taxonomic assignment was done using DADA2 against the SILVA 16S rRNA SSU database release 138 [42]. ASVs representing chloroplasts, mitochondria, and singletons were removed.

To test potential biases inherent to different DNA extraction methods and ensure statistical significance of the sequence data in terms of stratigraphy, we conducted one-way ANOSIM analysis with the Bray-Curtis index. Calculation of alpha and beta diversity was tested using ASV abundance matrices for all reads and rarefied to 2000 (Supplementary Data S1). Downstream statistical analyses were then performed with the standardized and non-rarefied dataset.

The Shannon index (alpha diversity), based on all 7975 ASVs, and a Canonical Correspondence Analysis (CCA), based on the 3000 most abundant ASVs and 21 explanatory geochemical variables, were computed using PAST v. 4.03 [43]. The significance of the canonical axes was tested via permutation (*N* = 999). To ensure robustness of the results, the CCA was cross-checked via a separate non-metric multidimensional scaling (NMDS) computed with all 7975 ASVs, and a principal component analysis (PCA) with the 21 geochemical parameters, using PAST v. 4.03.

To trace persistent taxa within microbial communities, we plotted two Venn diagrams from a total of 7331 ASVs (gravity core: 5 samples; core TDP-1A: 21 samples), respectively depicting archaeal and bacterial ASVs common to all 3 stratigraphic units (hereafter persistent ASVs) previously defined for core TDP-1A, using Venny v. 2.1.0 [44]. Relative abundances of common ASVs were plotted downcore as bar charts.

All partial 16S rRNA gene amplicon sequences were aligned using SINA online v.1.2.11 [45] and inserted into the SILVA 138.1 SSURef_NR99 tree, using the ARB parsimony algorithm, with the bacterial and archaeal filters enabled [46]. Three distinct Maximum Likelihood RAxML phylogenetic trees were then plotted in ARB, selecting the closest sequence matches to the inserted ASVs, to display phylogenetic affiliations of the 1000 most abundant ASVs, the ASVs common to all three stratigraphic units, and all ASVs identified in Unit 2, respectively.

## Results

### Bulk Sediment Reflects Depositional Conditions Along the Stratigraphy

**Fig. 2:**
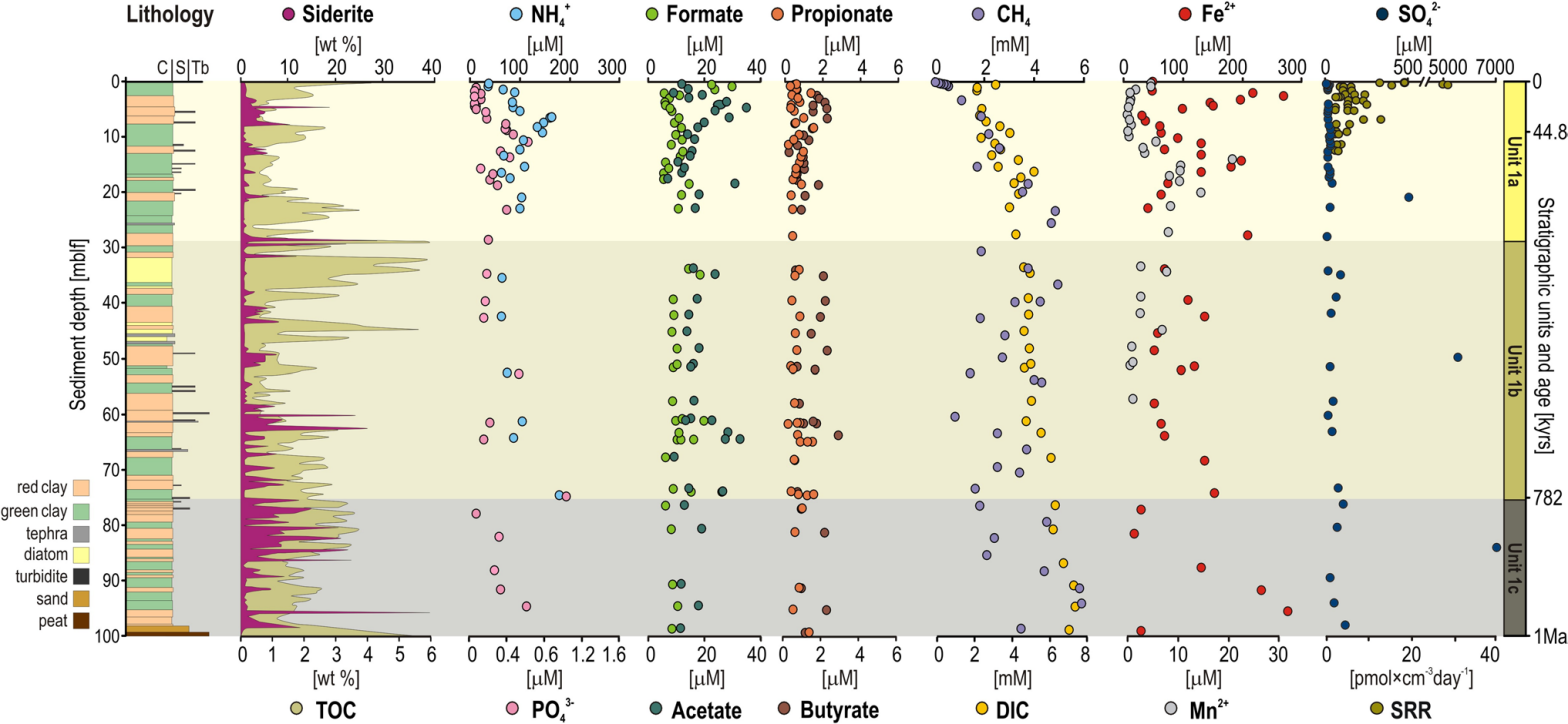
Downcore profiles for bulk sediment and pore water geochemistry. (From left to right) Lithology of core TPD-1A, siderite content and total organic carbon (TOC), pore water concentrations in ammonium (NH_4_^+^), phosphate (PO_4_^3−^), formate, acetate, propionate and butyrate, dissolved methane (CH_4_), dissolved inorganic carbon (DIC), ferrous iron (Fe^2+^), manganese (Mn^2+^), sulfate (SO_4_^2−^), and measured sulfate reduction rates (SRR). The figure is modified from [28]

The stratigraphic sequence consists of alternating red and green clay layers (Supplementary Fig. S1), which reflect different redox conditions at the SWI at the time of deposition [29, 33]. Although primary productivity in modern Lake Towuti is low, TOC values are in general higher in green clays (ca. 2 to 3 weight %), whereas red clays show an opposite tendency [30] with lower TOC (≤ 2% wt) but higher siderite (≥ 10% wt) concentrations (Fig. 2). TN concentrations covary with those of TOC, while TOC/TN values recorded an admixture of terrestrial and algal organic sources in link to sedimentation types and trophic states (Supplementary Fig. S2). Sporadic decreases in bulk organic content reflect turbidites, while phases of algal productivity (e.g., diatom oozes) account for increased TOC values.

From bottom to top, the stratigraphy exhibits maximum TOC values (Fig. 2) in the basal peat layer at the transition from Unit 2 (i.e., riverine sequence) to Unit 1c (i.e., shallow lacustrine sequence), followed by recurrent fluctuations in TOC and siderite contents related to red/green clay alternations [16, 29]. These observations are supported by a concomitant increase in the TOC/TN ratio, which is indicative of increased terrestrial OM (Supplementary Fig. S2). Decreased sedimentation in Unit 1b resulted in siderite-rich layers, with the occurrence of diatom oozes (45 to 30 mblf), which reflect a high productivity phase [34], likely with increased nutrient recycling at the SWI [29, 32]. Unit 1a exhibits wide variations in TOC values related to lake level highstands and lowstands due to climate- and tectonic-driven hydrological changes in the catchment, such as river capture events followed by evaporation during the Last Glacial Maximum [28, 47].

### Pore Water Geochemical Evolution During Early Diagenesis

Microbial activity significantly influences pore water geochemistry after deposition due to reductive processes [15]. Briefly, depletion of terminal electron acceptors (i.e., O_2_, NO_3_^−^, Mn^4+^, Fe^3+^, SO_4_^2−^) already occurs in the monimolimnion (Fig. 1B) and continues in the sediment (Fig. 2), according to the canonical chain of respiration types [48], with a transition into the fermentative zone between 5 and 10 cmblf [25].

Within the upper 10 m of sediment (Fig. 2), OM breakdown releases NH_4_^+^ and PO_4_^3−^ into the pore water, followed by microbial uptake and consumption. Similarly, VFA profiles (i.e., formate, acetate, propionate, butyrate) can be interpreted in terms of stepwise degradation of OM, as they are being produced as fermentative by-products and consumed as substrates, leading to rapid conversion in pore water [15, 36]. As dissolved inorganic carbon (DIC) and methane (CH_4_), the end products of VFA remineralization, continuously accumulate in the pore water and reach concentrations of up to 5 mM near the bottom of Unit 1a (20 mblf). Further downcore DIC concentrations increase more slowly but steadily, whereas CH_4_ concentrations are lower in turbidite beds (Fig. 2). Altogether, these pore water profiles highlight the dynamic behavior of organic solutes in Unit 1a, whereas steady concentrations characterize Unit 1b and Unit 1c. Sporadic variations in otherwise steady geochemical profiles are associated with turbidite events and specific lithologies (e.g., diatoms, tephra) [17].

In contrast, pore water concentrations in dissolved metals (Fig. 2) reflect the initial dissolution of reactive Fe-bearing phases (e.g., ferrihydrite) during reductive diagenesis [48]. Following their release into pore water, Fe^2+^ and Mn^2+^ are subsequently incorporated into authigenic phases as pore water becomes saturated with respect to siderite [31] and vivianite [32] in the upper 10 mblf and below 20 mblf, respectively.

### Sulfate Reduction Rates, Cell Density, and 16S rRNA Genes Decrease in the Subsurface

SO_4_^2−^ concentrations (< 12 μM) become rapidly depleted in the sediment due to the activity of sulfate-reducing bacteria (SRB), reaching levels at or below our detection limits around 12 mblf. Sulfate reduction rates (SRR) decrease progressively from ca. 30 pmol × cm^−3^ d^−1^ at the SWI to negligible levels at 12 mblf, the depth at which sulfate availability becomes limiting (Fig. 2). SRR measurements were therefore not carried out in deeper sediments. In Unit 1b and Unit 1c, there are two intervals bearing tephra layers (i.e., 51.5 and 87.5 mblf) that also show sulfate peaks (i.e., 5227.5 and 7010.8 μM) (Fig. 2).

**Fig. 3:**
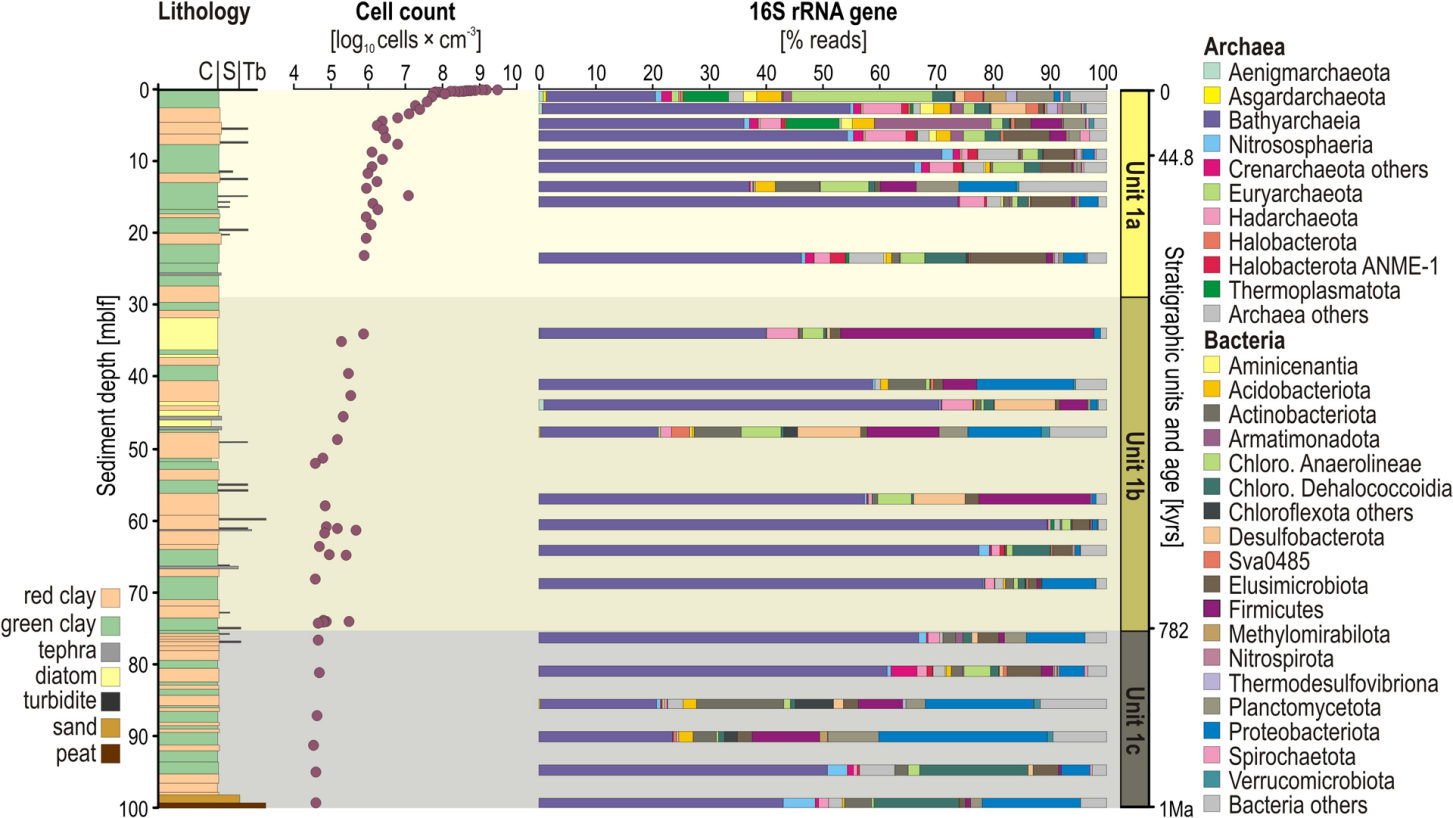
Cell densities and taxonomic diversity of microbial populations. (From left to right) Lithology of core TPD-1A, downcore profiles for microbial cell counts and relative abundance of 16S rRNA genes and their taxonomic classification at the phylum/class level

Total cell counts (Fig. 3) are highest at the SWI (i.e., log_10_ = 9.5 cells × cm^−^3). Depletion in pore water electron acceptors (i.e., Fe^3+^, SO_4_^2−^) in the upper 50 cmblf further translates into a steep decrease (i.e., 3 orders of magnitude) in cell counts (log_10_ = 6.3) down to 5 mblf into the fermentative zone. Deeper (ca. 15 mblf), cell counts decrease gradually to 5 and 4 log_10_ cells × cm^−^^3^ at the bottom of the core, which are similar to values previously reported for other ICDP sediment records from anoxic lakes [49].

Bioinformatic treatment of 16S rRNA gene sequences from 40 discrete samples resulted in 2,085,345 processed reads assigned to 7975 different ASVs, of which 2940 ASVs (~ 35%) were identified as Archaea and 5035 ASVs (~ 65%) as Bacteria. Results of the One-Way ANOSIM show that the parameter “DNA extraction method” did not affect sample distribution, while the parameter “stratigraphic units” proved statistically highly significant (Supplementary Fig. S3). Thus, taxonomic assignments reveal that the subsurface biosphere of Lake Towuti is differentially distributed along the sediment sequence (Fig. 3). Archaeal populations are predominantly represented by the phyla Bathyarchaeia, Hadarchaeota, and Nitrososphaeria, whereas most abundant bacterial phyla correspond to Chloroflexota (i.e., class Anaerolineae and Dehalococcoidia), Actinobacteria, Firmicutes (i.e., Bacillota) and Proteobacteria (i.e., Pseudomonadota).

Chloroflexota account for ca. 30% of total 16S rRNA reads at the SWI. Despite an initial decrease in the upper 0.5 mblf (> 10%), this phylum remains predominant in the taxonomic assemblage with depth, stabilizing at < 10% below 50 mblf. Desulfobacterota are also significant at the SWI (ca. 10–15%), but decrease consistently with SRR (Figs. 2 and 3), except in the vicinity of the intervals exhibiting increased pore water sulfate (51.5 and 87.5 mblf). Bathyarchaeia, which already make up the majority of the microbial assemblage at 0.5 mblf (40% of all reads), become even more prevalent (ca. 70%) at mid-depth of the core, reaching their highest relative abundance (88.4%) at 61.6 mblf (Fig. 3).

Variations in relative abundances of phyla alongside pore water geochemistry can be broadly interpreted in terms of functional guilds (e.g., iron reducers, sulfate reducers, fermenters, methanogens), which likely reflect different metabolic roles of the subsurface biosphere at increasing depths [25]. Phylogenetic analysis of the 1000 most abundant ASVs (Supplementary Fig. S4) supports such linkage between taxonomy and functions, and highlights specific taxa among Acidobacteriota as putative iron-reducing bacteria (FeRB), Desulfobacterota and Firmicutes as potential SRB and syntrophs, several Halobacterota and Euryarchaeota as putative methanogens, and mainly Chloroflexota (class Dehalococcoidia) and Bathyarchaeia as the main fermenters.

### Statistical Analyses of Microbial Community Structure

**Fig. 4:**
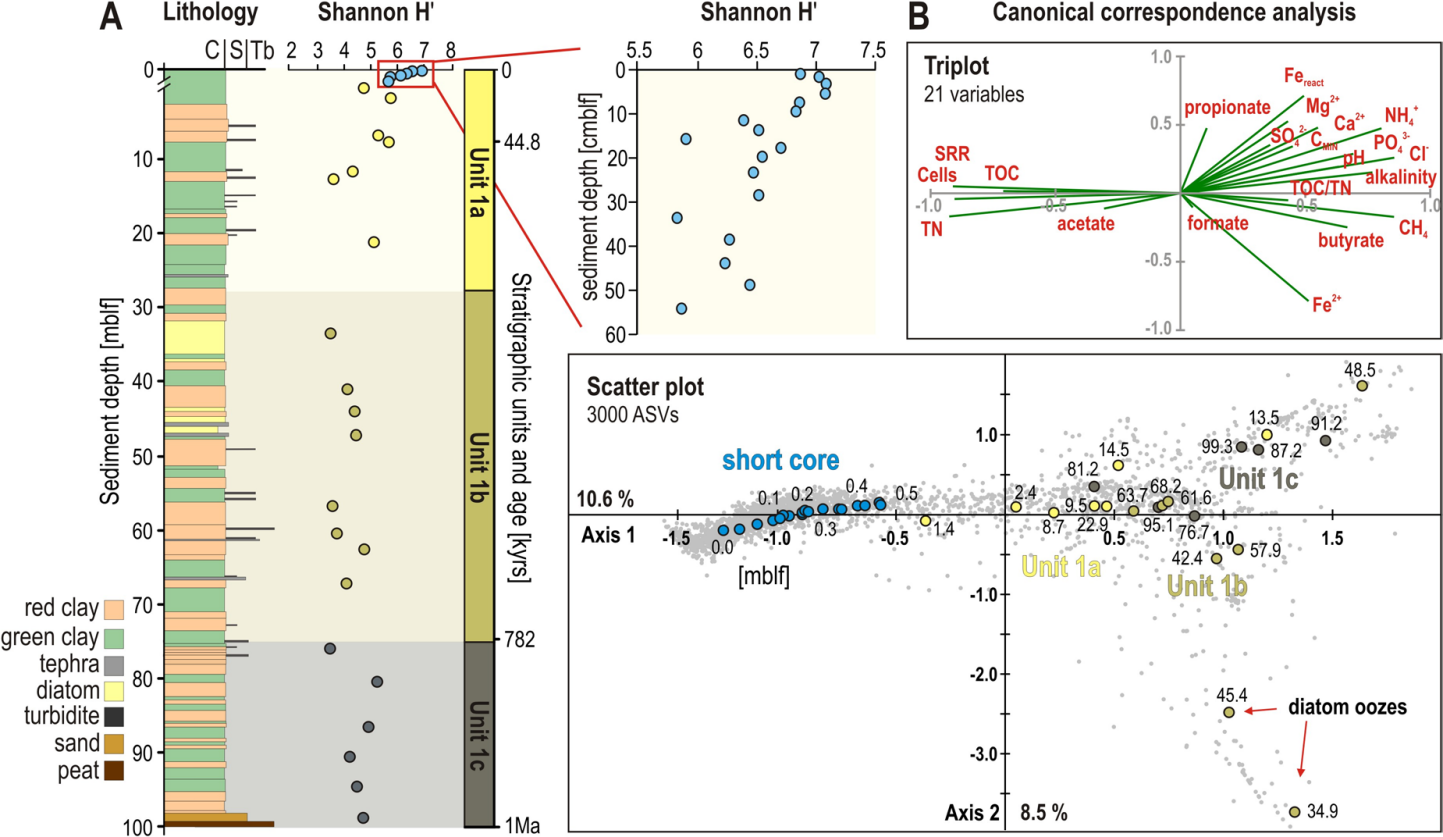
Alpha and beta diversity of microbial populations. **A** Downcore profile of alpha diversity shown as Shannon index values (H’), with close-up on the upper 60 cmblf. **B** Beta diversity based on canonical correspondence analysis (CCA) computed based on 21 environmental variables (triplot) and the 3000 most abundant ASVs (scatter plot). Samples (circles) and ASVs (dots) are partially distributed according to substrate depletion with sediment depth (Axis 1) and lithologies (Axis 2)

Calculation of alpha and beta diversity based on the full and rarefied sequence data produced comparable results (Supplementary Fig. S5). The Shannon index profile (i.e., 7975 ASVs) displays variations in alpha diversity across the lithologies composing the three stratigraphic units (Fig. 4). In the uppermost layer of Unit 1a (0–50 cmblf) wherein the Shannon index is highest, microbial populations appear to be most diverse but experience drastic reductions with depth. Based on total cell counts, SRR and 16S rRNA gene taxonomy (Figs. 2, 3, to 4), such high alpha diversity is inferred to correspond to an active SRB community, thriving in freshly deposited sediments. The Shannon index gradually decreases from 0 to 20 mblf as the taxonomic assemblage shifts to mainly fermentative archaea (Fig. 3), while OM remineralization and conversion of pore water VFAs proceed in parallel (Fig. 2). Unit 1b (20–70 mblf) harbors less diverse microbial populations that are almost exclusively composed of Bathyarchaeia (ca. 90% of all reads), reflecting a deep biosphere assembly under nutrient-depleted conditions. Despite shifts in taxonomic diversity in the vicinity of diatom oozes (i.e.., 45–47, 32–37 mblf) and sulfate peaks associated with tephra layers (i.e., 48, 80 mblf), the Shannon index in Unit 1b exhibits low but stable values (Figs. 2, 3, 4). In contrast, Unit 1c (70–100 mblf) is characterized by an increased microbial diversity (H’ = ca. 6).

The CCA plot depicts a distinct pattern of beta diversity and clusters ASVs in relation to stratigraphic units (Fig. 4). The 21 parameters selected as explanatory variables reflect differences in lithology and geochemistry. Axis 1, which accounts for 10.6% of the variance, divides explanatory variables into two groups: TOC, TN, SRR, and high cell counts on the left-hand side, metabolic end-products in pore water with low cell densities on the right-hand side, with VFAs at the center of the triplot. Axis 1 is thus consistent with substrate depletion. Axis 2, which accounts for 8.5% of the variance, seems to separate samples according to lithologies potentially characteristic of each stratigraphic unit, thereby helping to distinguish between siderite-rich (i.e., C_MIN_, Fe_react_) and OM-rich (i.e., TOC/TN, CH_4_) layers. Samples from the upper 50 cmblf are grouped together and connected to those of Unit 1a by a linear trend. These samples are characterized by higher microbial diversity and density. Most samples across Unit 1a to Unit 1c spread linearly along axis 1 and cluster tightly together along axis 2, reflecting gradual substrate depletion in similar lithologies. Specific samples more distal to axis 2 correspond to specific lithologies (i.e., diatom oozes, tephra-associated sulfate peaks).

Such clustering of environmental parameters and stratigraphic distribution of ASVs were also observed in the PCA and NMDS plots (Supplementary Figs. S6-S7), respectively.

### Persistent ASVs Become Prevalent in Deep Sediments

**Fig. 5:**
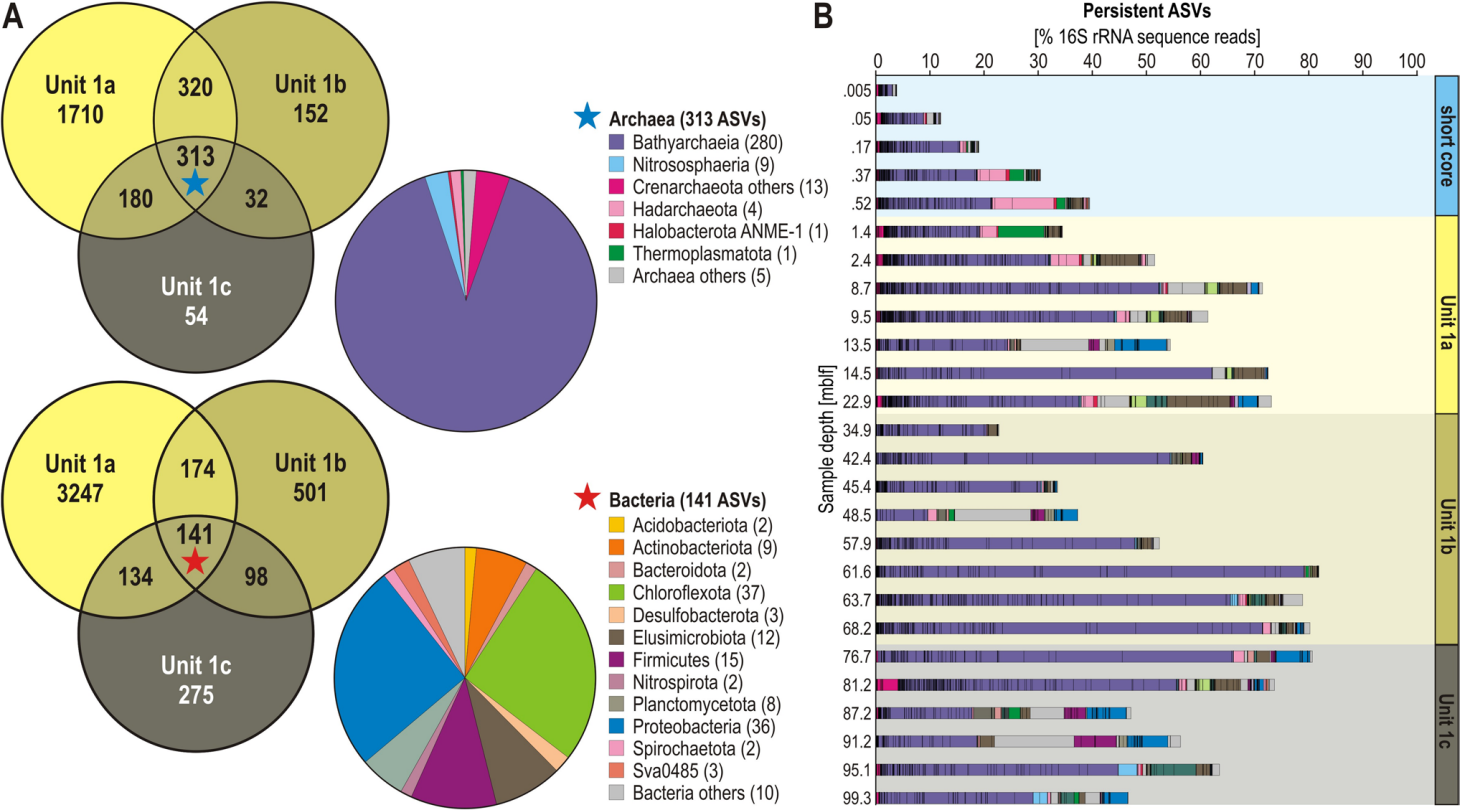
Distribution, taxonomy and relative abundance of the persistent microbial community. **A** Venn diagrams depicting taxonomic richness in terms of the number of archaeal (**top**) and bacterial (**bottom**) ASVs across the three stratigraphic units. The stars at the center signify persistent ASVs that are common to all three stratigraphic units and whose respective taxonomic richness is detailed in the pie charts. **B** Relative abundances of persistent ASVs colored according to their taxonomic assignments at the phylum level along the gravity core (5 selected samples), Unit 1a, 1b and 1c. The numbers in brackets in the legend indicate the number of ASVs identified in the corresponding phylum

The 313 persistent archaeal ASVs (Fig. 5) apparently derive from a deep biosphere assembly, which is composed of selective taxa pre-adapted to substrate depletion. They include numerous bathyarchaeal ASVs whose relative abundance clearly increases with sediment depth (Fig. 5B).

The 141 persistent bacterial ASVs (Fig. 5) constitute a heterogenous assemblage including taxa belonging to Firmicutes (i.e., Bacillota) and Proteobacteriota (i.e., Pseudomonadota). Detailed phylogenetic analysis of persistent ASVs (Supplementary Figs. S8-S10) confirms the prevalence of Bathyarchaeia (280 ASVs) selected from a deep biosphere assembly associated with Chloroflexota (37 ASVs) and diverse Bacteria known to persist via dormancy (e.g., Proteobacteriota) and resting stages (e.g., Actinobacteriota, Firmicutes, Planctomycetota).

### Unit 2 Is Consistent with Terrestrial Inputs and Soil-Derived Microbes

Unit 2 contains various lithologies (Supplementary Fig. S1) that indicate less lacustrine and more fluvial and deltaic sedimentation. The transition from Unit 2 to Unit 1 marks a shift from riverine environments to a wetland, recorded by a thick peat layer, and then to a shallow lake [17, 29]. The CCA plot, comprising the ASVs identified in Unit 2, distinctly separates them from those of the overlying three units (Supplementary Fig. S11), suggesting a different microbial assemblage. Similarly, the additional PCA plot locates samples from Unit 2 in a quadrant previously assigned to organic-rich deep sediments and dormant cells (Supplementary Fig. S6). Phylogenetic analysis of the microbial assemblages associated with Unit 2 revealed higher relative abundance of specific bacterial phyla compared to archaeal phyla (Supplementary Fig. S12), e.g., Proteobacteria (class Alpha- and Gammaproteobacteria), Firmicutes (i.e., Bacillota), Planctomycetota, Actinobacteriota, and Chloroflexota. Finally, residues of plant material encrusted by siderite were identified in some organic-rich layers of Unit 2 (Supplementary Fig. S13), revealing terrestrial input that underwent substantial microbial degradation.

Altogether, this suggests that microbial populations in Unit 2 are largely composed of allochthonous taxa introduced with terrestrial inputs, and lesser autochthonous taxa involved in different stages of sedimentary OM remineralization.

## Discussion

### Lacustrine Subsurface Biosphere Linked to Short- and Long-Term Environmental Changes

Geochemical indicators can document past environmental conditions in the lacustrine realm where the sedimentation regime is highly dynamic over time. These indicators can also characterize the geochemical environment housing subsurface microbial communities; however, a link between lacustrine deposition of variable lithologies and the density and structure of subsurface microbial populations has only been reported for Holocene (12 ka) to Late Pleistocene (~ 129 ka) sediments [9, 50–52]. The most significant environmental factors indirectly exerting control on microbial community structure in lake sediments are annual precipitation, temperature and vegetation cover [53, 54] as they determine in turn water column stratification, salinity, and organic sources, thus defining the deposition of substrates in terms of electron acceptors and donors available to microorganisms in the subsurface. Although lake floors generally shelter high phylogenetic diversity and active metabolisms at the time of deposition [55, 56], geochemical redox conditions radically shift as sediments accumulate, exerting selection on microbial communities evolving in a closed trophic network [57]. Gradual substrate depletion during burial is expected to select for a deep biosphere community, which tends to follow the geochemical gradient that develops during early diagenesis and deviate from the stratigraphic record [11, 23, 58].

Despite the fact that tropical lakes experience little overall seasonal variations [59], Lake Towuti’s alternation of green and red clay along the sedimentary sequence reflects specific environmental fluctuations, e.g., iron inflows [18, 33], depth of the oxycline [29], redox conditions at the SWI [20, 22]. Lake Towuti’s subsurface biosphere appeared dense, diverse, and active near the SWI, but consistently declined over the upper 10 mblf of Unit 1a (Figs. 2, 3, 4), layering functional microbial guilds according to the gradual depletion in electron acceptors and donors (Figs. 1–2). Processes of early diagenesis proceeded differentially in red and green clay, as they contain more electron acceptors and donors, respectively, leading to increased mineralization of ferric substrates or continued OM remineralization to produce either siderite or methane [15, 33]. In the lower part (> 10 mblf) of Unit 1a, microbial communities appeared to be less influenced by depositional conditions but confronted with substrate limitation, resulting in a majority of Bathyarchaeia unrelated to stratigraphy (Fig. 3).

In contrast, periods of different depositional regimes [18, 29, 47] resulted in significantly different alpha and beta diversity (Fig. 4; Supplementary Figs. S4, S5 and S7), for instance when lake isolation promoted internal nutrient recycling (Unit 1b) or active fluvial connection frequent terrestrial inflows (Unit 1c). We found that specific lithological horizons significantly influenced microbial community composition by either promoting sulfate reduction (tephras at 23, 48, and 81 mblf), syntrophic fermentation (diatom oozes at 35 and 43 mblf), or redeposition of terrestrial microbes (peat and shallower lake deposits from 99 to 82 mblf), thereby increasing the corresponding relative abundances of Desulfobacterota, Firmicutes, Proteobacteriota and Actinobacteriota populations (Fig. 3; Supplementary Data S1).

Over its 1 Ma geological timescale, Lake Towuti experienced significant climate- and tectonic-driven environmental shifts [29, 47], resulting in variable vegetation cover [26], weathering in the catchment [18], primary productivity [34], water level [16], and the deposition of mineral and organic material on the lake floor [33, 60]. While short-term lake fluctuations promoted specific microbial processes in individual green and red layers during early diagenesis, long-term hydrological dynamics of Lake Towuti definitively controlled depositional conditions, the ensuing lithologies exerting selection on microbial diversity and composition of 16S rRNA genes (Figs. 3–4). Overall, these findings highlight how long-term history of environmental and depositional conditions can shape microbial landscape at the lake floor, have a lasting impact on the composition of microbial populations [10, 11] and/or preservation of their sedimentary DNA [26, 61], and promote different biogeochemical cycles and diagenetic processes in the sediment [15, 31, 32].

### Ferruginous Conditions and Substrate Depletion Select for a Deep Biosphere Assembly

Lake Towuti’s ferruginous bottom waters and sediments are characterized by a rapid depletion in terminal electron acceptors (Fig. 2), creating a strong redox gradient that shapes microbial community composition (Fig. 3).

In iron- and sulfate-reducing sediments, labile OM is broken down [62], initially releasing NH_4_^+^ and PO_4_^3−^ to the pore water as by-products (Fig. 2). Due to fermentation having a lower energy yield, cells tend to become much smaller and accumulate metabolites in the fermentative zone. Under substrate depletion, these metabolites (e.g., VFAs) become a major source of reactive carbon in the fermentative zone [63] as they can be remineralized stepwise via syntrophy [64]. The downcore profiles of VFAs demonstrate such dynamic turnover (Fig. 2; Supplementary Fig. S2), with the release and conversion of lactate and formate, and successively butyrate, acetate and propionate [65], achieving complete remineralization to DIC and methane in pore water (5 mM) at 20 mblf. Alongside depletion in electron acceptors in the pore water, microbial cell densities decreased by three orders of magnitude (log_10_ = 9.6 to 6.5) from the SWI down to 4 mblf (Fig. 3) then, after SRR had ceased (6 to 8 mblf), continuously by two orders of magnitude into the deep fermentative zone (log_10_ = 6.5 to 4.5) that reaches to the bottom of the core. Despite active carbon transformations along the upper 20 m of sediment, we suggest that microbial cells deeply buried in sediments persist at turnover rates extending over decades, even hundreds of years [66, 67].

Depth succession of putative functional guilds was inferred from pore water geochemical profiles, decreasing cell densities and shifts in microbial community composition (Figs. 2–3), with substrate-based selection into the fermentative zone. The functional guilds FeRB [68] and SRB [69] were tentatively assigned to ASVs among the phyla Acidobacteriota, Desulfobacterota and Firmicutes (Supplementary Fig. S4), whose relative abundances were higher in the upper 50 cmblf (Fig. 4). However, metabolic versatility is a common feature among FeRB and SRB, and strict primary fermenters were predicted among Chloroflexota (class Anaerolineae) and Elusimicrobiota. Syntrophic interactions [70, 71] with hydrogenotrophic methanogens [36] were inferred from taxa among Desulfobacterota and Firmicutes associated with Euryarchaeota, Halobacterota and Thermoplasmatota (Supplementary Fig. S4), which appeared more abundant in intervals where VFAs were actively remineralized (Figs. 2–3). Secondary fermenters are found among acetogens that can utilize C1 compounds (e.g., formate, methanol, methylamines) and excrete acetate or produce energy from it [72], e.g., Firmicutes, Hadarchaeota [73]. However, as recalcitrant and oxidized OM accumulates with fermentation by-products [74], acetate is also incorporated into biomass, alike in clades of Dehalococcoidia [24] and Bathyarchaeia [25], while H_2_ and CO_2_ remain the main energy source.

Bathyarchaeia are predicted to have the metabolic potential to harness redox energy from reduced sulfur [75], explaining how they can prevail after the sulfate reduction zone, namely by recycling sulfides in the fermentative zone [25]. Furthermore, their capability to access a wide range of refractory organic compounds (i.e., carbohydrate-active enzymes) as substrates [76] or solely utilize H_2_ and CO_2_ as mixotrophic (homo)acetogens demonstrates an adaptation to long-term survival under energy limitation [77]. Similarly, (homo)acetogenic Dehalococcoidia can recycle highly oxidized organic compounds (i.e., necromass) to perform secondary fermentations and persist under substrate-limited conditions [24, 78]. Because (homo)acetogens can produce fermentative hydrogen and fix CO_2_ via the energy-conserving Wood–Ljungdahl pathway [79, 80], they tend to outcompete hydrogenotrophic methanogens under substrate-limiting conditions [81], and by analogy in deep sedimentary settings.

With DIC accumulating down to 100 mblf (Fig. 2), our findings demonstrate that Bathyarchaeia play a key role in carbon transformations in the lacustrine subsurface [10, 58, 76], suggesting the ability to sustain their metabolism over a million-year confinement.

### Deep Subsurface Niche Partitioning Selects Persistent Taxa alongside Microbial Seed Banks

Below 20 mblf (Unit1a), the viability of the deep biosphere could not be unequivocally demonstrated. Continuously low cell densities, steady geochemical profiles, and microbial communities essentially composed of “microbial dark matter” [82] highly suggest that deeply buried cells maintain low metabolic rates, whereas specific subpopulations may be dead, dormant or only found as resting stages [83]. Microbial communities found at cell densities as low as 5–4 log_10_ cells × cm^−^^3^ might not have sufficient energy to grow and reach cell division, thus only maintain “housekeeping” metabolism [6, 84, 85]. Some terrestrial microorganisms (Supplementary Figs. S11-S12) sporadically transported into the lake via the fluvial system (e.g., turbidites, deltaic inflows) may have sufficient metabolic versatility to adapt to conditions in the water column or at the SWI, but usually enter dormancy [83, 86] or produce resting stages [87] in the subsurface.

Based on the ASV core assemblage identified in all three stratigraphic units (Fig. 5), the admixture of prokaryotic DNA extracted from subsurface sediments indicates a deep biosphere assemblage likely descended from surface members [10, 14, 88] whose relative abundance significantly increased with sediment depth (Fig. 5A–B). The uppermost Unit 1a supported a dynamic microbial community revolving around active processes of sulfate reduction and stepwise fermentation, ultimately resulting in complete remineralization to CH_4_ and DIC at 20 mblf. At such depths, the persistent ASV community contributed nearly to half of the microbial diversity, highlighting selection according to substrate partitioning during OM remineralization. In the subsequent Unit 1b, reduced sedimentation promoted internal nutrient recycling and resulted in high primary productivity [34] and organic-rich layers, which favored Desulfobacterota and Firmicutes populations (Fig. 3), shifting the assembly balance between persistent and transient ASVs (Fig. 5A). In Unit 1c, some of the ASVs assigned to Bathyarchaeia and common to all three units became extremely predominant with depth of burial, increasing from ca. 5 to 80% total reads from surface to deep sediments (Fig. 5B). In contrast, persistent bacterial ASVs (Fig. 5) constituted a heterogenous assemblage including hypothetic resting stages [86], some introduced with terrigenous inflows, and subsisting at different relative abundances depending on local geochemical conditions (i.e., ferric inputs, organic content, pore water sulfate). Because nucleic acids represent a preferential substrate in nutrient-depleted sediments [39], we assumed that extracellular DNA from dead cells could not accumulate with burial [39] unlike dormant cells and resting stages.

Thus, similar to previous ICDP studies on lacustrine environments [11, 49, 89], the density and taxonomic diversity of the subsurface biosphere shows a coherent link with sediment stratigraphy and composition. As changes in sediment input, depositional conditions, and redox conditions at the SWI resulted in specific lithologies (e.g., peat, diatom ooze, tephra, turbidite), sediment substrates and their geochemical evolution exerted differential selection on microbial populations, which apparently adapted to the corresponding stratigraphic niches and thus to the corresponding geochemical conditions. These local modifications to the subsurface ecosystem illustrate the interplay between depositional conditions, substrate composition, and microbial community structure.

Although adaptation to deep ferruginous settings cannot be inferred from 16S rRNA gene data, environmental sequences closely affiliated with persistent Bathyarchaeia (and Dehalococcoidia) encompass isolation sources (Supplementary Figs. S9-S10) potentially compatible with a chronosequence of disconnection from the sea (i.e., hydrothermal vent, marine subseafloor, estuary, river, wetland, and lake) and a relatively recent marine-terrestrial divide [90]. According to molecular clock estimates on fish populations [91, 92], the Malili Lake System disconnected from the sea some 1.9 Ma ago. This raised questions about the ability of marine Bathyarchaeia and Dehaloccoidia to disperse, colonize, and undergo adaptive selection for ferruginous settings on the million-year timescale [93]. Interesting divergences between marine and terrestrial clades [94] appear to include specific metabolic functions, such as the absence of the *mcrA* (i.e., methanogenesis), *aprAB* and *dsrAB* (i.e., dissimilatory sulfate reduction) genes in lacustrine Bathyarchaeia [25] and Dehalococcoidia [24], respectively.

## Conclusions

Our study represents the first comprehensive characterization of Lake Towuti’s deep subsurface biosphere, spanning a one-million-year lacustrine sedimentary sequence. Sedimentological analyses combined with geochemical profiles and 16S rRNA gene taxonomy demonstrate how depositional conditions influence microbial community composition over short- and long-term burial. As environmental changes assumed to control the lake’s redox stratification dictated the substrates deposited, their availability in the subsurface shaped interactions between microbial functional guilds in terms of electron donors and acceptors, driving differential early diagenetic processes during burial. The dominance of Bathyarchaeia in Lake Towuti’s sediment and their ability to sustain metabolism over geologic timescales reveal them as key players of organic carbon transformations in the lacustrine subsurface. The interplay between environmental changes, microbial life, and substrate mineralization in subsurface sediments is essential to understanding long-term biogeochemical cycles in lacustrine sedimentary ecosystems.

## Supplementary Information

Below is the link to the electronic supplementary material.

Supplementary file1 (XLSX 3213 KB)

Supplementary file2 (PDF 9740 KB)

## Data Availability

All raw 16S rRNA gene sequencing data are publicly available on the European Nucleotide Archive (ENA) under project accession no. PRJEB85713 and PRJEB66721 (https://www.ebi.ac.uk/). The geochemical datasets (#861437, #908080, #934401) are publicly available from the PANGAEA® Data Publisher for Earth & Environmental Science (https://doi.pangaea.de/) under DOI no. 10.1594/PANGAEA.861437, 10.1594/PANGAEA.908080), and 10.1594/PANGAEA.934401.

## References

[1] Beaver RC, Neufeld JD (2024) Microbial ecology of the deep terrestrial subsurface. ISME J 18:wrae091. 10.1093/ismejo/wrae091 38780093 10.1093/ismejo/wrae091 PMC11170664

[2] Cario A, Oliver GC, Rogers KL (2019) Exploring the deep marine biosphere: Challenges, innovations, and opportunities. Front Earth Sci 7:225. 10.3389/feart.2019.00225

[3] Magnabosco C, Lin L-H, Dong H et al (2018) The biomass and biodiversity of the continental subsurface. Nature Geosci 11:707–717. 10.1038/s41561-018-0221-6

[4] Edwards KJ, Bach W, McCollom TM (2005) Geomicrobiology in oceanography: microbe–mineral interactions at and below the seafloor. Trends Microbiol 13:449–456. 10.1016/j.tim.2005.07.005 16054363 10.1016/j.tim.2005.07.005

[5] Jørgensen BB, Boetius A (2007) Feast and famine - microbial life in the deep-sea bed. Nat Rev Microbiol 5:770–781. 10.1038/nrmicro1745 17828281 10.1038/nrmicro1745

[6] Orsi WD, Schink B, Buckel W, Martin WF (2020) Physiological limits to life in anoxic subseafloor sediment. FEMS Microbiol Rev 44:219–231. 10.1093/femsre/fuaa004 32065239 10.1093/femsre/fuaa004 PMC7269680

[7] Kallmeyer J, Pockalny R, Adhikari RR et al (2012) Global distribution of microbial abundance and biomass in subseafloor sediment. P Natl A Sci USA 109:16213–16216. 10.1073/pnas.1203849109 10.1073/pnas.1203849109 PMC3479597 22927371

[8] Colwell FS, D’Hondt S (2013) Nature and extent of the deep biosphere. Rev Mineral Geochem 75:547–574. 10.2138/rmg.2013.75.17

[9] Ariztegui D, Thomas C, Vuillemin A (2015) Present and future of subsurface biosphere studies in lacustrine sediments through scientific drilling. Int J Earth Sci 104:1655–1665. 10.1007/s00531-015-1148-4

[10] Vuillemin A, Ariztegui D, Horn F et al (2018) Microbial community composition along a 50 000-year lacustrine sediment sequence. FEMS Microbiol Ecol 94:fiy029. 10.1093/femsec/fiy029 29471361 10.1093/femsec/fiy029 PMC5905624

[11] Thomas C, Francke A, Vogel H et al (2020) Weak influence of paleoenvironmental conditions on the subsurface biosphere of Lake Ohrid over the last 515 ka. Microorganisms 8:1736. 10.3390/microorganisms8111736 33167482 10.3390/microorganisms8111736 PMC7716225

[12] Berg JS, Lepine M, Laymand E et al (2022) Ancient and modern geochemical signatures in the 13,500-year sedimentary record of Lake Cadagno. Front Earth Sci 9:754888. 10.3389/feart.2021.754888

[13] Wurzbacher C, Fuchs A, Attermeyer K et al (2017) Shifts among Eukaryota, Bacteria, and Archaea define the vertical organization of a lake sediment. Microbiome 5:41. 10.1186/s40168-017-0255-9 28388930 10.1186/s40168-017-0255-9 PMC5385010

[14] Walsh EA, Kirkpatrick JB, Rutherford SD et al (2016) Bacterial diversity and community composition from seasurface to subseafloor. ISME J 10:979–989. 10.1038/ismej.2015.175 26430855 10.1038/ismej.2015.175 PMC4796937

[15] Vuillemin A, Morlock M, Paskin A et al (2023) Authigenic minerals reflect microbial control on pore waters in a ferruginous analogue. Geochem Persp Let 28:20–26. 10.7185/geochemlet.2339

[16] Ulfers A, Hesse K, Zeeden C et al (2021) Cyclostratigraphy and paleoenvironmental inference from downhole logging of sediments in tropical Lake Towuti, Indonesia. J Paleolimnol 65:377–392. 10.1007/s10933-020-00171-9

[17] Russell JM, Bijaksana S, Vogel H et al (2016) The Towuti Drilling Project: paleoenvironments, biological evolution, and geomicrobiology of a tropical Pacific lake. Sci Dril 21:29–40. 10.5194/sd-21-29-2016

[18] Morlock MA, Vogel H, Nigg V et al (2019) Climatic and tectonic controls on source-to-sink processes in the tropical, ultramafic catchment of Lake Towuti, Indonesia. J Paleolimnol 61:279–295. 10.1007/s10933-018-0059-3

[19] Zegeye A, Bonneville S, Benning LG et al (2012) Green rust formation controls nutrient availability in a ferruginous water column. Geology 40:599–602. 10.1130/G32959.1

[20] Bauer KW, Byrne JM, Kenward P et al (2020) Magnetite biomineralization in ferruginous waters and early Earth evolution. Earth Planet Sc Lett 549:116495. 10.1016/j.epsl.2020.116495

[21] Poulton SW, Canfield DE (2011) Ferruginous conditions: A dominant feature of the ocean through Earth’s history. Elements 7:107–112. 10.2113/gselements.7.2.107

[22] Vuillemin A, Friese A, Alawi M et al (2016) Geomicrobiological features of ferruginous sediments from Lake Towuti. Indonesia Front Microbiol 7:1007. 10.3389/fmicb.2016.01007 27446046 10.3389/fmicb.2016.01007 PMC4928248

[23] Vuillemin A, Horn F, Friese A et al (2018) Metabolic potential of microbial communities from ferruginous sediments. Environ Microbiol 20:4297–4313. 10.1111/1462-2920.14343 29968357 10.1111/1462-2920.14343

[24] Vuillemin A, Ruiz-Blas F, Yang S et al (2024) Taxonomic and functional partitioning of Chloroflexota populations under ferruginous conditions at and below the sediment-water interface. FEMS Microbiol Ecol 100:fiae140. 10.1093/femsec/fiae140 39384533 10.1093/femsec/fiae140 PMC11650866

[25] Ruiz-Blas F, Bartholomäus A, Yang S et al (2024) Metabolic features that select for Bathyarchaeia in modern ferruginous lacustrine subsurface sediments. ISME Commun 4:ycae112. 10.1093/ismeco/ycae112 39660009 10.1093/ismeco/ycae112 PMC11631310

[26] Ekram MA-E, Campbell M, Kose SH et al (2024) A 1 Ma sedimentary ancient DNA (sedaDNA) record of catchment vegetation changes and the developmental history of tropical Lake Towuti (Sulawesi, Indonesia). Geobiology 22:e12599. 10.1111/gbi.12599 38745401 10.1111/gbi.12599

[27] Ekram MA-E, Wuchter C, Bijaksana S et al (2025) A Quaternary Sedimentary Ancient DNA (sedaDNA) Record of Fungal-Terrestrial Ecosystem Dynamics in a Tropical Biodiversity Hotspot (Lake Towuti, Sulawesi, Indonesia). Microorganisms 13:1005. 10.3390/microorganisms13051005 40431178 10.3390/microorganisms13051005 PMC12113726

[28] Vuillemin A, Mayr C, Schuessler JA et al (2023) A one-million-year isotope record from siderites formed in modern ferruginous sediments. GSA Bull 135:504–522. 10.1130/B36211.1

[29] Russell JM, Vogel H, Bijaksana S et al (2020) The late quaternary tectonic, biogeochemical, and environmental evolution of ferruginous Lake Towuti. Indonesia Palaeogeogr Palaeocl 556:109905. 10.1016/j.palaeo.2020.109905

[30] Ordoñez L, Vogel H, Sebag D et al (2019) Empowering conventional Rock-Eval pyrolysis for organic matter characterization of the siderite-rich sediments of Lake Towuti (Indonesia) using End-Member Analysis. Org Geochem 134:32–44. 10.1016/j.orggeochem.2019.05.002

[31] Vuillemin A, Wirth R, Kemnitz H et al (2019) Formation of diagenetic siderite in modern ferruginous sediments. Geology 47:540–544. 10.1130/G46100.1

[32] Vuillemin A, Friese A, Wirth R et al (2020) Vivianite formation in ferruginous sediments from Lake Towuti, Indonesia. Biogeosciences 17:1955–1973. 10.5194/bg-17-1955-2020

[33] Sheppard RY, Milliken RE, Russell JM et al (2021) Iron mineralogy and sediment color in a 100 m drill core from Lake Towuti, Indonesia reflect catchment and diagenetic conditions. Geochem Geophy Geosy 22:e2020GC009582. 10.1029/2020GC009582

[34] Ageli MK, Hamilton PB, Bramburger AJ et al (2022) Benthic-pelagic state changes in the primary trophic level of an ancient tropical lake. Palaeogeogr Palaeocl 594:110937. 10.1016/j.palaeo.2022.110937

[35] Friese A, Kallmeyer J, Axel Kitte J et al (2017) A simple and inexpensive technique for assessing contamination during drilling operations: A simple and inexpensive technique. Limnol Oceanogr-Meth 15:200–211. 10.1002/lom3.10159

[36] Friese A, Bauer K, Glombitza C et al (2021) Organic matter mineralization in modern and ancient ferruginous sediments. Nat Commun 12:2216. 10.1038/s41467-021-22453-0 33850127 10.1038/s41467-021-22453-0 PMC8044167

[37] Glombitza C, Pedersen J, Røy H, Jørgensen BB (2014) Direct analysis of volatile fatty acids in marine sediment porewater by two-dimensional ion chromatography-mass spectrometry: Analysis of volatile fatty acids in marine porewater. Limnol Oceanogr-Meth 12:455–468. 10.4319/lom.2014.12.455

[38] Kallmeyer J, Smith DC, Spivack AJ, D’Hondt S (2008) New cell extraction procedure applied to deep subsurface sediments: Cell extraction of deep subsurface sediments. Limnol Oceanogr-Meth 6:236–245. 10.4319/lom.2008.6.236

[39] Vuillemin A, Horn F, Alawi M et al (2017) Preservation and significance of extracellular DNA in ferruginous sediments from Lake Towuti. Indonesia Front Microbiol 8:1440. 10.3389/fmicb.2017.01440 28798742 10.3389/fmicb.2017.01440 PMC5529349

[40] Martin M (2011) Cutadapt removes adapter sequences from high-throughput sequencing reads. EMBnet J 17:10–12. 10.14806/ej.17.1.200

[41] Callahan BJ, McMurdie PJ, Rosen MJ et al (2016) DADA2: High-resolution sample inference from Illumina amplicon data. Nat Methods 13:581–583. 10.1038/nmeth.3869 27214047 10.1038/nmeth.3869 PMC4927377

[42] Quast C, Pruesse E, Yilmaz P et al (2013) The SILVA ribosomal RNA gene database project: improved data processing and web-based tools. Nucleic Acids Res 41:D590–D596. 10.1093/nar/gks1219 23193283 10.1093/nar/gks1219 PMC3531112

[43] Hammer O, Harper DAT, Ryan PD (2001) PAST: Paleontological statistics software package for education and data analysis. Palaeont Electr 4:1–9. https://doc.rero.ch/record/15326/files/PAL_E2660.pdf. Accessed 6 Dec 2024

[44] Venny 2.1.0. https://bioinfogp.cnb.csic.es/tools/venny/. Accessed 6 Dec 2024

[45] Pruesse E, Quast C, Knittel K et al (2007) SILVA: a comprehensive online resource for quality checked and aligned ribosomal RNA sequence data compatible with ARB. Nucleic Acids Res 35:7188–7196. 10.1093/nar/gkm864 17947321 10.1093/nar/gkm864 PMC2175337

[46] Ludwig W, Strunk O, Westram R et al (2004) ARB: a software environment for sequence data. Nucleic Acids Res 32:1363–1371. 10.1093/nar/gkh293 14985472 10.1093/nar/gkh293 PMC390282

[47] Russell JM, Vogel H, Konecky BL et al (2014) Glacial forcing of central Indonesian hydroclimate since 60,000 y B.P. P Natl A Sci USA 111:5100–5105. 10.1073/pnas.1402373111 10.1073/pnas.1402373111 PMC3986195 24706841

[48] Banfield JF, Nealson KH (1997) Geomicrobiology: Interactions between microbes and minerals. Reviews in Mineralogy Volume 35. Mineralogical Society of America, De Gruyter, Chantilly

[49] Kallmeyer J, Grewe S, Glombitza C, Kitte JA (2015) Microbial abundance in lacustrine sediments: a case study from Lake Van, Turkey. Int J Earth Sci 104:1667–1677. 10.1007/s00531-015-1219-6

[50] Dong H, Jiang H, Yu B, Liu X (2010) Impacts of environmental change and human activity on microbial ecosystems on the Tibetan Plateau, NW China. GSAT 4–10. 10.1130/GSATG75A.1

[51] Vuillemin A, Ariztegui D (2013) Geomicrobiological investigations in subsaline maar lake sediments over the last 1500 years. Quaternary Sci Rev 71:119–130. 10.1016/j.quascirev.2012.04.011

[52] Nam Y-D, Sung Y, Chang H-W et al (2008) Characterization of the depth-related changes in the microbial communities in Lake Hovsgol sediment by 16S rRNA gene-based approaches. J Microbiol 46:125–136. 10.1007/s12275-007-0189-1 18545961 10.1007/s12275-007-0189-1

[53] Møller TE, van der Bilt WGM, Roerdink DL, Jørgensen SL (2020) Microbial community structure in Arctic lake sediments reflect variations in Holocene climate conditions. Front Microbiol 11:1520. 10.3389/fmicb.2020.01520 32903319 10.3389/fmicb.2020.01520 PMC7396534

[54] Li M, Li Q, Wang S et al (2024) The diversity and biogeography of bacterial communities in lake sediments across different climate zones. Environ Res 263:120028. 10.1016/j.envres.2024.120028 39307222 10.1016/j.envres.2024.120028

[55] Newton RJ, Jones SE, Eiler A et al (2011) A guide to the natural history of freshwater lake bacteria. Microbiol Mol Biol R 75:14–49. 10.1128/mmbr.00028-10 10.1128/MMBR.00028-10 PMC3063352 21372319

[56] Vuillemin A, Coolen MJL, Kallmeyer J et al (2023) Bacterial and archaeal DNA from lake sediments. In: Capo E, Barouillet C, Smol JP (eds) Tracking Environmental Change Using Lake Sediments, vol 6. Sedimentary DNA. Springer International Publishing, Cham, pp 85–151

[57] Gralka M, Szabo R, Stocker R, Cordero OX (2020) Trophic interactions and the drivers of microbial community assembly. Curr Biol 30:R1176–R1188. 10.1016/j.cub.2020.08.007 33022263 10.1016/j.cub.2020.08.007

[58] Rodriguez P, Berg JS, Deng L et al (2025) Persistent functional and taxonomic groups dominate an 8,000-year sedimentary sequence from Lake Cadagno. Switzerland Front Microbiol 16:1504355. 10.3389/fmicb.2025.1504355 39990142 10.3389/fmicb.2025.1504355 PMC11843047

[59] Pu T, Haffner GD, Crowe SA, Katsev S (2025) Stratification stability of tropical lakes and their sensitivity to climate. Limnol Oceanogr. 10.1002/lno.70055

[60] Morlock MA, Vogel H, Russell JM et al (2021) Quaternary environmental changes in tropical Lake Towuti, Indonesia, inferred from end-member modelling of X-ray fluorescence core-scanning data. J Quaternary Sci 36:1040–1051. 10.1002/jqs.3338

[61] Capo E, Monchamp M-E, Coolen MJL et al (2022) Environmental paleomicrobiology: using DNA preserved in aquatic sediments to its full potential. Environ Microbiol 24:2201–2209. 10.1111/1462-2920.15913 35049133 10.1111/1462-2920.15913 PMC9304175

[62] Glombitza C, Stockhecke M, Schubert CJ et al (2013) Sulfate reduction controlled by organic matter availability in deep sediment cores from the saline, alkaline Lake Van (Eastern Anatolia, Turkey). Front Microbiol 4:209. 10.3389/fmicb.2013.00209 23908647 10.3389/fmicb.2013.00209 PMC3725400

[63] Glombitza C, Egger M, Røy H, Jørgensen BB (2019) Controls on volatile fatty acid concentrations in marine sediments (Baltic Sea). Geochim Cosmochim Ac 258:226–241. 10.1016/j.gca.2019.05.038

[64] Morris BEL, Henneberger R, Huber H, Moissl-Eichinger C (2013) Microbial syntrophy: interaction for the common good. FEMS Microbiol Rev 37:384–406. 10.1111/1574-6976.12019 23480449 10.1111/1574-6976.12019

[65] Stams AJM, Plugge CM (2009) Electron transfer in syntrophic communities of anaerobic bacteria and archaea. Nat Rev Microbiol 7:568–577. 10.1038/nrmicro2166 19609258 10.1038/nrmicro2166

[66] Braun S, Mhatre SS, Jaussi M et al (2017) Microbial turnover times in the deep seabed studied by amino acid racemization modelling. Sci Rep 7:5680. 10.1038/s41598-017-05972-z 28720809 10.1038/s41598-017-05972-z PMC5516024

[67] Mhatre SS, Kaufmann S, Marshall IPG et al (2019) Microbial biomass turnover times and clues to cellular protein repair in energy-limited deep Baltic Sea sediments. FEMS Microbiol Ecol 95:fiz068. 10.1093/femsec/fiz068 31095297 10.1093/femsec/fiz068

[68] Weber KA, Achenbach LA, Coates JD (2006) Microorganisms pumping iron: anaerobic microbial iron oxidation and reduction. Nat Rev Microbiol 4:752–764. 10.1038/nrmicro1490 16980937 10.1038/nrmicro1490

[69] Muyzer G, Stams AJM (2008) The ecology and biotechnology of sulphate-reducing bacteria. Nat Rev Microbiol 6:441–454. 10.1038/nrmicro1892 18461075 10.1038/nrmicro1892

[70] Kendall MM, Liu Y, Boone DR (2006) Butyrate- and propionate-degrading syntrophs from permanently cold marine sediments in Skan Bay, Alaska, and description of *Algorimarina butyrica* gen. nov., sp. nov. FEMS Microbiol Lett 262:107–114. 10.1111/j.1574-6968.2006.00380.x 16907746 10.1111/j.1574-6968.2006.00380.x

[71] Liu Y, Conrad R, Yao T et al (2017) Change of methane production pathway with sediment depth in a lake on the Tibetan plateau. Palaeogeogr Palaeocl 474:279–286. 10.1016/j.palaeo.2016.06.021

[72] Wolfe AJ (2005) The acetate switch. Microbiol Mol Biol R 69:12–50. 10.1128/mmbr.69.1.12-50.2005 10.1128/MMBR.69.1.12-50.2005 PMC1082793 15755952

[73] Baker BJ, Saw JH, Lind AE et al (2016) Genomic inference of the metabolism of cosmopolitan subsurface Archaea, Hadesarchaea. Nat Microbiol 1:1–9. 10.1038/nmicrobiol.2016.2 10.1038/nmicrobiol.2016.2 27572167

[74] Shang H (2023) A generic hierarchical model of organic matter degradation and preservation in aquatic systems. Commun Earth Environ 4:1–10. 10.1038/s43247-022-00667-437325084

[75] Feng X, Wang Y, Zubin R, Wang F (2019) Core metabolic features and hot origin of Bathyarchaeota. Engineering 5:498–504. 10.1016/j.eng.2019.01.011

[76] Zhou Z, Pan J, Wang F et al (2018) Bathyarchaeota: globally distributed metabolic generalists in anoxic environments. FEMS Microbiol Rev 42:639–655. 10.1093/femsre/fuy023 29790926 10.1093/femsre/fuy023

[77] Hou J, Wang Y, Zhu P et al (2023) Taxonomic and carbon metabolic diversification of Bathyarchaeia during its coevolution history with early Earth surface environment. Sci Adv 9:eadf5069. 10.1126/sciadv.adf5069 37406125 10.1126/sciadv.adf5069 PMC10321748

[78] Vuillemin A, Kerrigan Z, D’Hondt S, Orsi WD (2020) Exploring the abundance, metabolic potential and gene expression of subseafloor Chloroflexi in million-year-old oxic and anoxic abyssal clay. FEMS Microbiol Ecol 96:fiaa223. 10.1093/femsec/fiaa223 33150943 10.1093/femsec/fiaa223 PMC7688785

[79] Ragsdale SW, Pierce E (2008) Acetogenesis and the Wood-Ljungdahl pathway of CO_2_ fixation. Biochim Biophys Acta 1784:1873–1898. 10.1016/j.bbapap.2008.08.012 18801467 10.1016/j.bbapap.2008.08.012 PMC2646786

[80] Wiechmann A, Ciurus S, Oswald F et al (2020) It does not always take two to tango: “Syntrophy” via hydrogen cycling in one bacterial cell. ISME J 14:1561–1570. 10.1038/s41396-020-0627-1 32203116 10.1038/s41396-020-0627-1 PMC7242416

[81] Karekar S, Stefanini R, Ahring B (2022) Homo-acetogens: Their metabolism and competitive relationship with hydrogenotrophic methanogens. Microorganisms 10:397. 10.3390/microorganisms10020397 35208852 10.3390/microorganisms10020397 PMC8875654

[82] Rinke C, Schwientek P, Sczyrba A et al (2013) Insights into the phylogeny and coding potential of microbial dark matter. Nature 499:431–437. 10.1038/nature12352 23851394 10.1038/nature12352

[83] McDonald MD, Owusu-Ansah C, Ellenbogen JB et al (2024) What is microbial dormancy? Trends Microbiol 32:142–150. 10.1016/j.tim.2023.08.006 37689487 10.1016/j.tim.2023.08.006

[84] LaRowe DE, Amend JP (2015) Power limits for microbial life. Front Microbiol 6:718. 10.3389/fmicb.2015.00718 26236299 10.3389/fmicb.2015.00718 PMC4502533

[85] Vuillemin A, Vargas S, Coşkun Ö et al (2020) Atribacteria reproducing over millions of years in the Atlantic abyssal subseafloor. mBio 11:e01937-20. 10.1128/mBio.01937-20 33024037 10.1128/mBio.01937-20 PMC7542362

[86] Lennon JT, Jones SE (2011) Microbial seed banks: the ecological and evolutionary implications of dormancy. Nat Rev Microbiol 9:119–130. 10.1038/nrmicro2504 21233850 10.1038/nrmicro2504

[87] Paul C, Filippidou S, Jamil I et al (2019) Chapter Three - Bacterial spores, from ecology to biotechnology. In: Gadd GM, Sariaslani S (eds) Advances in Applied Microbiology. Academic Press, pp 79–111 10.1016/bs.aambs.2018.10.002 30798805

[88] Starnawski P, Bataillon T, Ettema TJG et al (2017) Microbial community assembly and evolution in subseafloor sediment. P Natl A Sci USA 114:2940–2945. 10.1073/pnas.1614190114 10.1073/pnas.1614190114 PMC5358386 28242677

[89] Vuillemin A, Ariztegui D, Lücke A et al (2014) Paleoenvironmental conditions define current sustainability of microbial populations in Laguna Potrok Aike sediments, Argentina. Aquat Sci 76:101–114. 10.1007/s00027-013-0317-4

[90] Ruff SE, de Angelis IH, Mullis M et al (2024) A global comparison of surface and subsurface microbiomes reveals large-scale biodiversity gradients, and a marine-terrestrial divide. Sci Adv 10:eadq0645. 10.1126/sciadv.adq0645 39693444 10.1126/sciadv.adq0645 PMC11654699

[91] Stelbrink B, Stöger I, Hadiaty RK et al (2014) Age estimates for an adaptive lake fish radiation, its mitochondrial introgression, and an unexpected sister group: Sailfin silversides of the Malili Lakes system in Sulawesi. BMC Evol Biol 14:94. 10.1186/1471-2148-14-94 24886257 10.1186/1471-2148-14-94 PMC4029975

[92] Vaillant JJ, Haffner GD, Cristescu ME (2011) The Ancient Lakes of Indonesia: Towards Integrated Research on Speciation. Integr Comp Biol 51:634–643. 10.1093/icb/icr101 21856735 10.1093/icb/icr101

[93] Bradley JA (2025) Microbial dormancy as an ecological and biogeochemical regulator on Earth. Nat Commun 16:3909. 10.1038/s41467-025-59167-6 40280922 10.1038/s41467-025-59167-6 PMC12032139

[94] Takeuchi M, Komai T, Hanada S et al (2009) Bacterial and Archaeal 16S rRNA Genes in Late Pleistocene to Holocene Muddy Sediments from the Kanto Plain of Japan. Geomicrobiol J 26:104–118. 10.1080/01490450802662355

