# Supplementary material for: The Deep Subsurface Biosphere and its Substrates Along a One-Million-Year Ferruginous Lake Archive
Source: Microb Ecol. 2025 Jun 3;88(1):58. doi: 10.1007/s00248-025-02559-4 (PMC12134053; doi:10.1007/s00248-025-02559-4)
Supplement: Supplementary file 2 — Supplementary file2 (PDF 9740 KB) [file 248_2025_2559_MOESM2_ESM.pdf]

Supplementary Information

The Deep Subsurface Biosphere and its Substrates along a One-Million-Year Ferruginous Lake Archive

Fatima Ruiz-Blas[1], André Friese[1], Alexander Bartholomaüs[1], Cynthia Henny[2], James M. Russell[3], Jens Kallmeyer[1] and Aurèle Vuillemin[1*]

[1] GFZ Helmholtz Centre for Geosciences, Section Geomicrobiology, Telegrafenberg, 14473 Potsdam, Germany

[2] Research Center for Limnology and Water Resources, National Research and Innovation Agency (BRIN), Republic of Indonesia, Cibinong, 16911, Jawa Barat, Indonesia

[3] Department of Earth, Environmental, and Planetary Sciences, Brown University, 324 Brook Street, Providence, RI, USA

Content

Supplementary Figures

- **Supplementary Figure S1.** High-resolution scan images of core sections from borehole TDP-1B

- **Supplementary Figure S2.** Downcore profiles for TN, TOC/TN ratios, and lactate concentrations

- **Supplementary Figure S3.** One-Way ANOSIM analyses for "DNA extraction" and "stratigraphic units"

- **Supplementary Figure S4.** 16S rRNA gene phylogenetic tree of the 1000 most abundant ASVs

- **Supplementary Figure S5.** Alpha and beta diversity based on full and rarified datasets

- **Supplementary Figure S6.** Principal component analysis (PCA), with and without samples from Unit 2

- **Supplementary Figure S7**. Non-metric multidimensional scaling (NMDS)

- **Supplementary Figure S8.** 16S rRNA gene phylogenetic tree of ASVs common to Unit 1a, 1b and 1c

- **Supplementary Figure S9.** 16S rRNA gene phylogenetic tree of persistent Bathyarchaeia ASVs

- **Supplementary Figure S10.** 16S rRNA gene phylogenetic tree of persistent Chloroflexota ASVs

- **Supplementary Figure S11.** Canonical correspondence analysis (CCA) including samples from Unit 2

- **Supplementary Figure S12.** Bar charts and 16S rRNA gene phylogenetic tree of ASVs from Unit 2

- **Supplementary Figure S13.** SEM images of plant residues incrusted by siderite from Unit 2

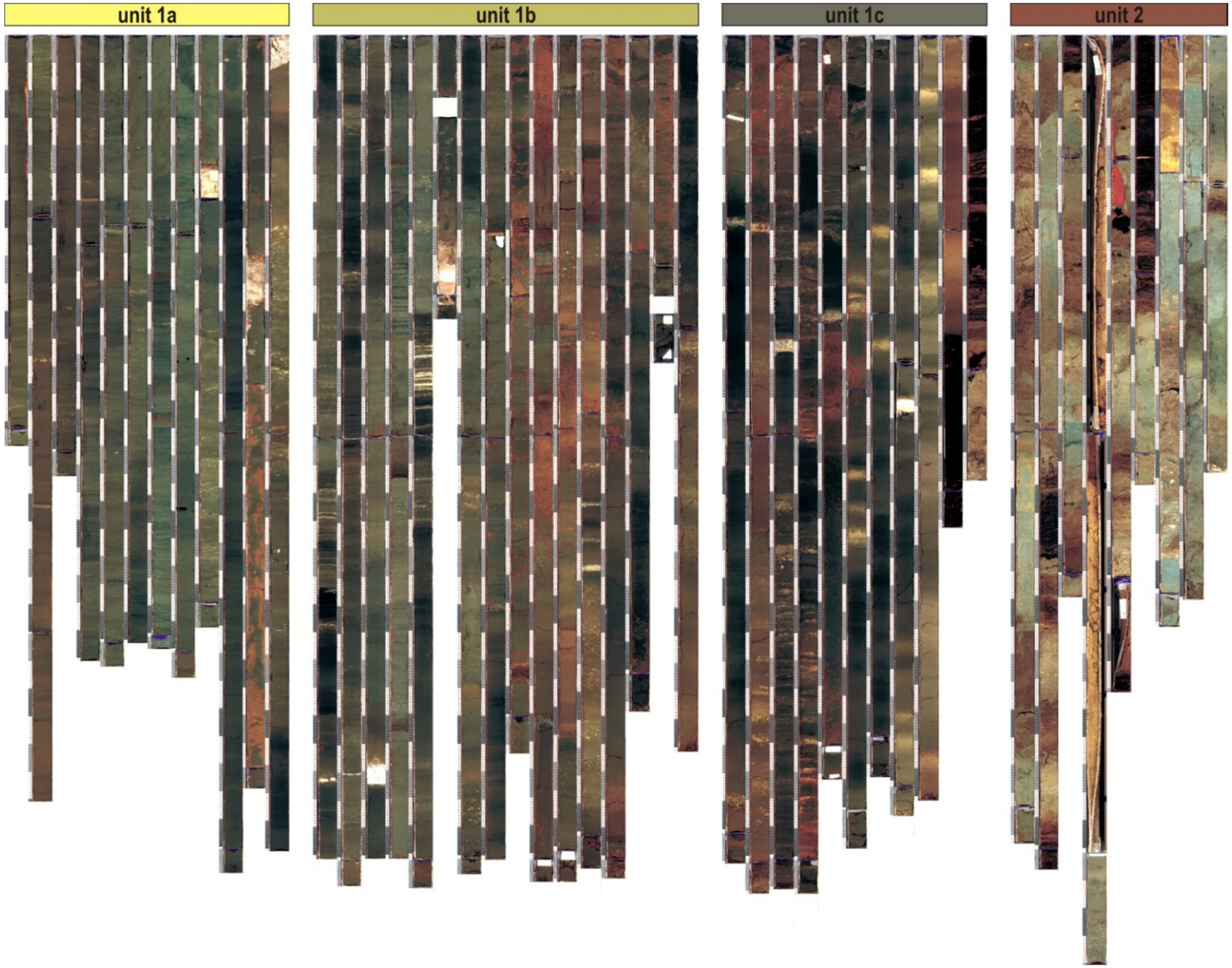

Supplementary Figure S1. Scan images of hydraulic cores from borehole TDP-1B. (From left to right) High-resolution scan images of core sections TDP-TOW15-1B-1H-1 (0.7 mblf) to TDP-TOW15-1B-50A-22 (120.5 mblf) are displayed according to the stratigraphic units to which they belong. Borehole TDP-1B is located at 156 m water depth.

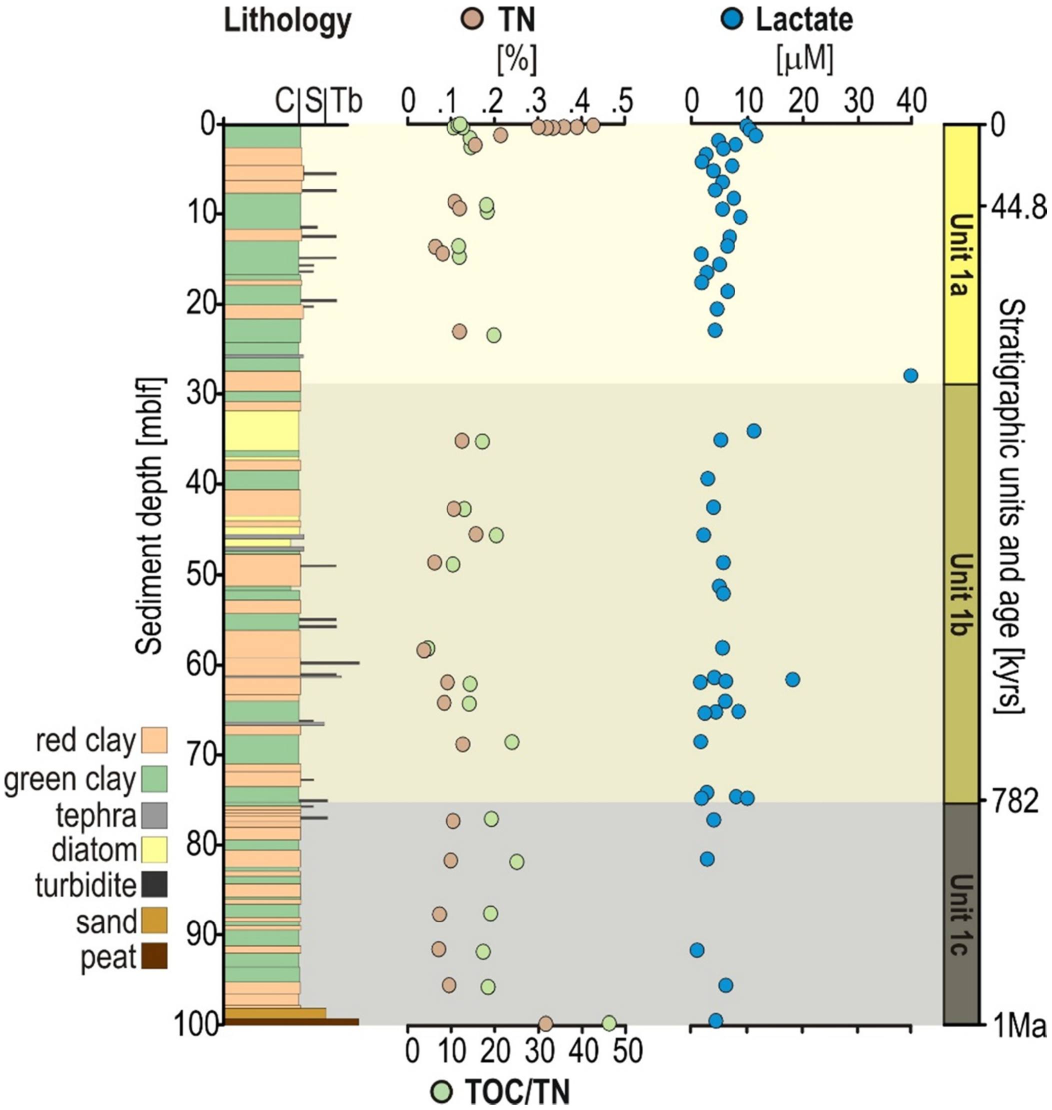

Supplementary Figure S2. Additional downcore profiles for bulk sediment and pore water. (From left to right) Total nitrogen (TN) in [weight %] and TOC/TN ratio; and pore water lactate concentrations [µM].

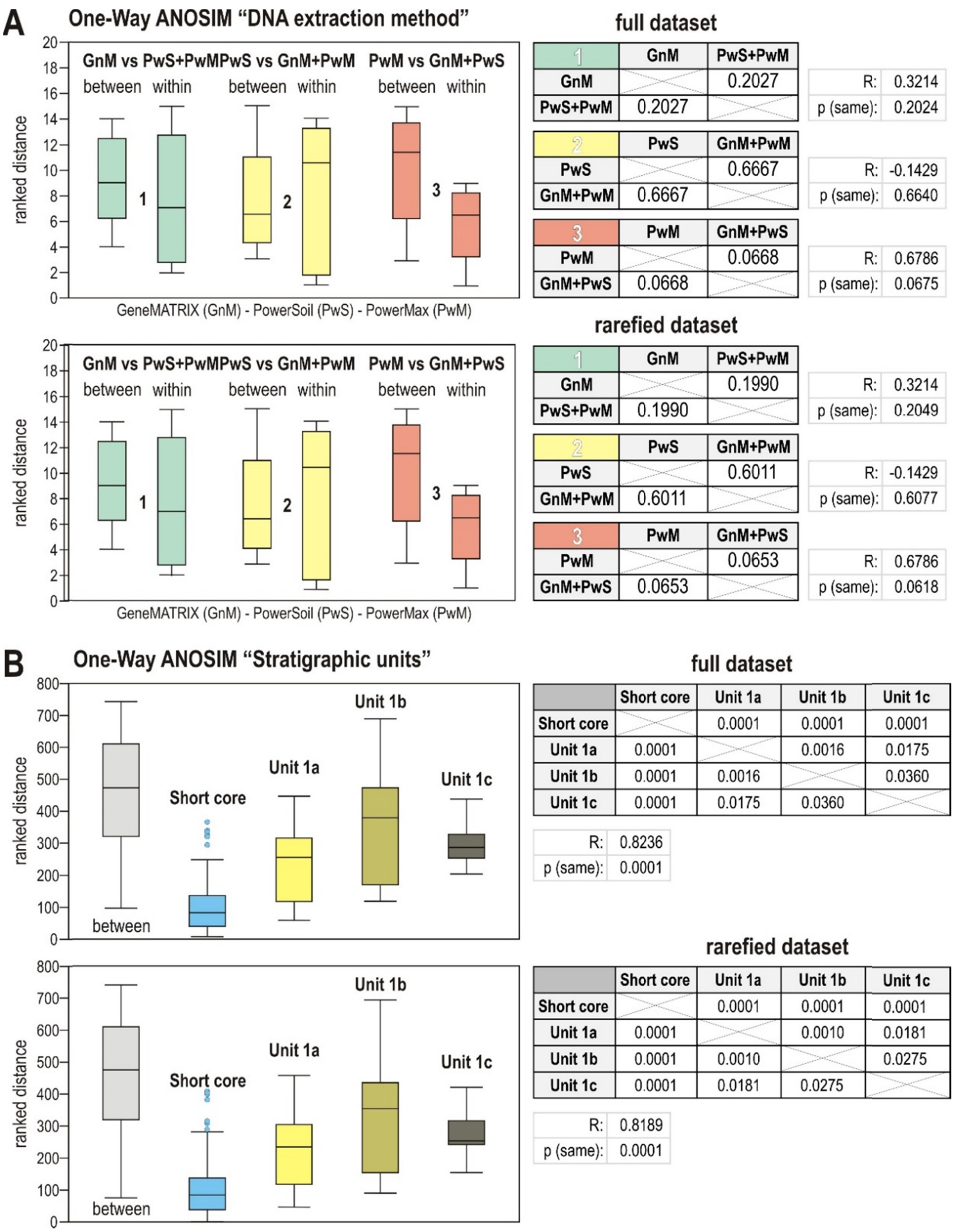

Supplementary Figure S3. One-Way ANOSIM analysis applied to test the parameters "DNA extraction method" and "stratigraphic units" using the complete and rarefied datasets. (A) Any potential bias in sequence data related to the use of three different DNA extraction kits (i.e. GeneMATRIX, PowerSoil, PowerMax) was tested via One-Way ANOSIM analysis (Bray Curtis index) with the full **(top)** and rarefied **(bottom)** dataset, using six samples from 0.5 to 9.5 mblf, i.e. two samples per DNA extraction kit. **(B)** The same approach was applied to test statistical significance of the parameter "stratigraphic units" in the distribution of the full **(top)** and rarefied **(bottom)** sequence data. Potential biases inherent to DNA extraction methods could be ruled out, and an interpretation of sequence data in terms of stratigraphy proved statistically significant.

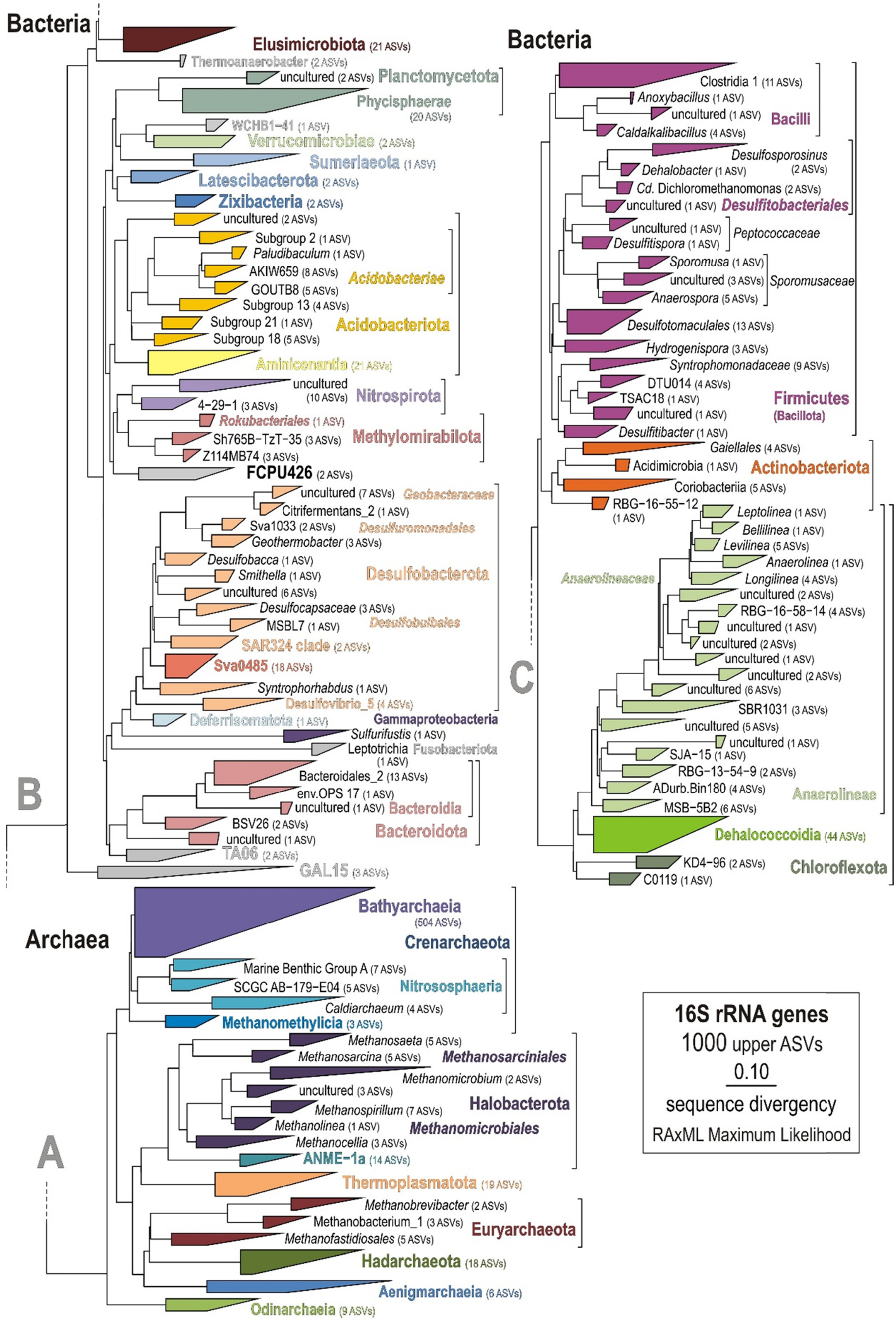

Supplementary Figure S4. Phylogenetic tree of 16S rRNA genes (V4 hypervariable region) for the 1000 most abundant amplicons sequenced in this study. Boldface types signify cultivated species and sequence accession numbers to the rRNA SSU database release 138. Partial 16S rRNA gene amplicons (500 bps) were inserted in the SILVA NR99 reference tree on ARB, applying the ARB Parsimony algorithm with the bacterial and archaeal filters.

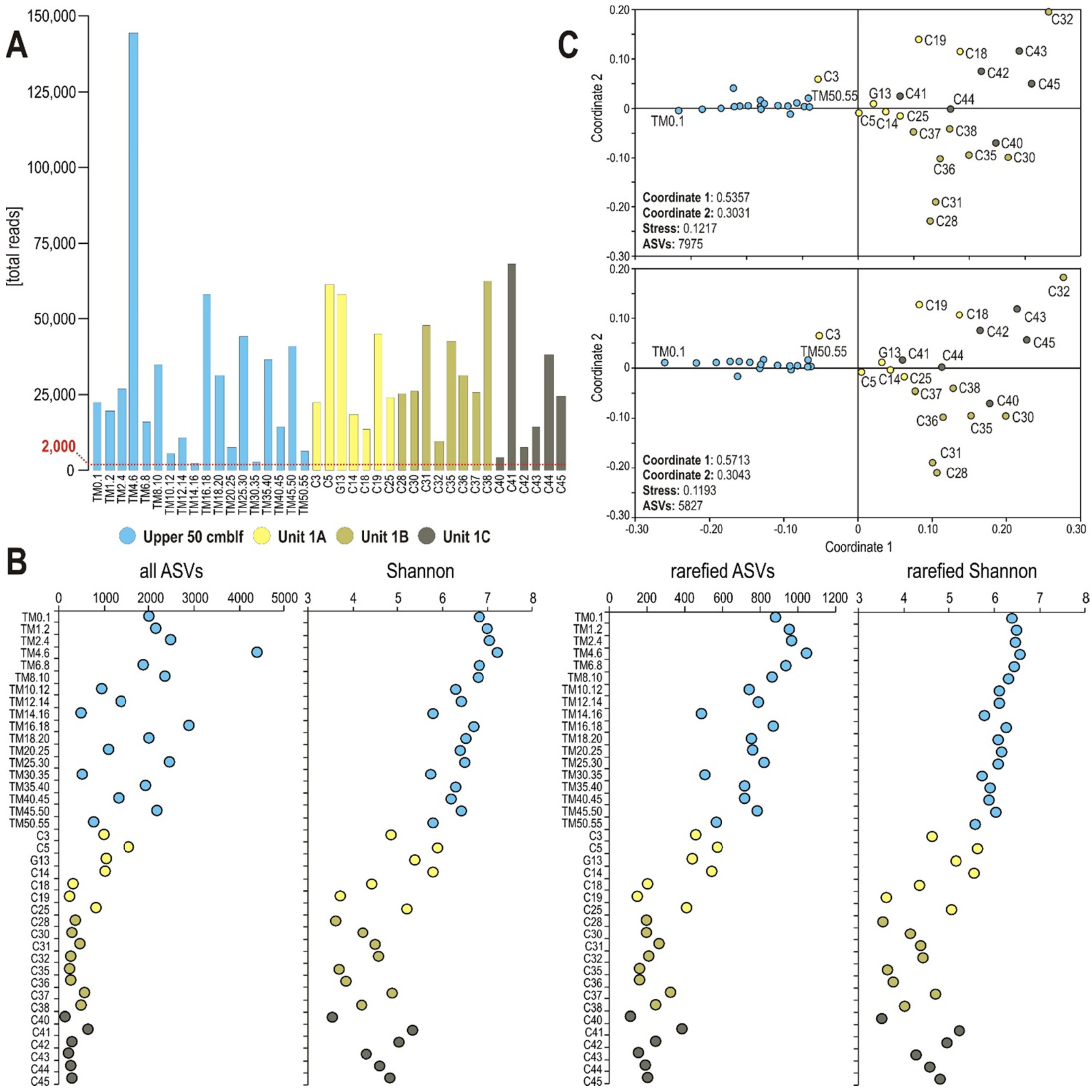

Supplementary Figure S5. Comparison of alpha and beta diversity calculated based on full and rarified datasets. (A) Sequencing depths per sample, expressed as total number of 16S rRNA amplicon reads. Dataset rarefaction is based on a cut-off value of 2000 reads per sample **(B)** Total number of ASVs and corresponding Shannon index values based on all (**left**) and rarefied (**right**) reads. **(C)** Non-metric multidimensional scaling (NMDS) based on 7975 and 5827 ASVs calculated with the full (**top**) and rarefied (**bottom**) dataset, respectively.

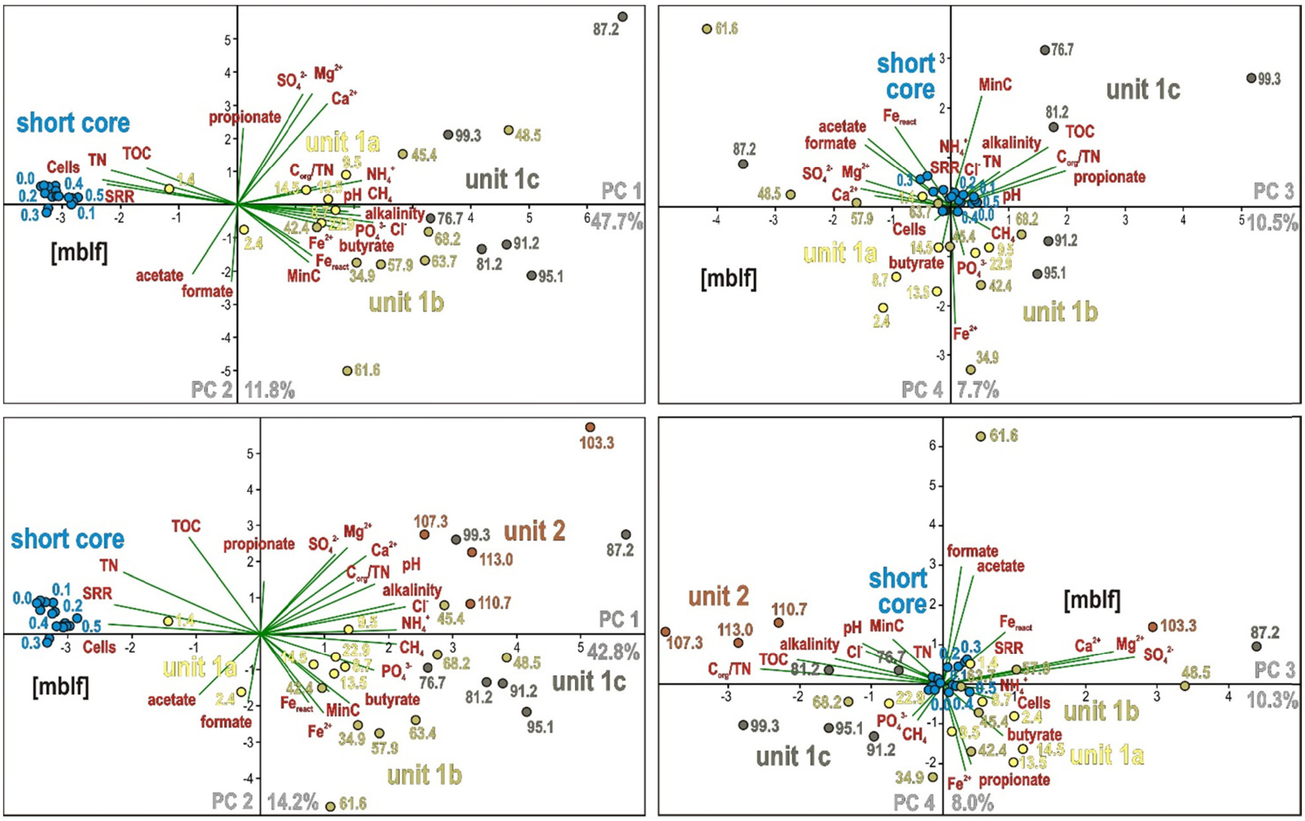

Supplementary Figure S6. Principal component analysis computed with and without samples from Unit 2. (Top) Principal component analysis (PCA) conducted with 21 explanatory variables on 39 samples, including a short gravity core (18 samples), and stratigraphic Unit 1a (7 samples), Unit 1b (8 samples), and Unit 1c (6 samples). The graph on the left depicts PC1 (47.7%) and PC2 (11.8%), which are consistent with the decrease in density and activity of the subsurface biosphere and pore water evolution with depth (PC1), and bulk-sediment-related parameters, such as the organic and siderite content (PC2). The graph on the right depicts PC3 (10.5%) and PC4 (7.7%), displaying concentric distribution of samples as a function of depth. **(Bottom)** PCA conducted with 21 explanatory variables on 43 samples, including 4 samples from fluvio-deltaic Unit 2 that cluster in the upper right quarter of the graph. PC1 to PC4 account for 42.8, 14.2, 10.3 and 8.0 % of the variance explained, respectively.

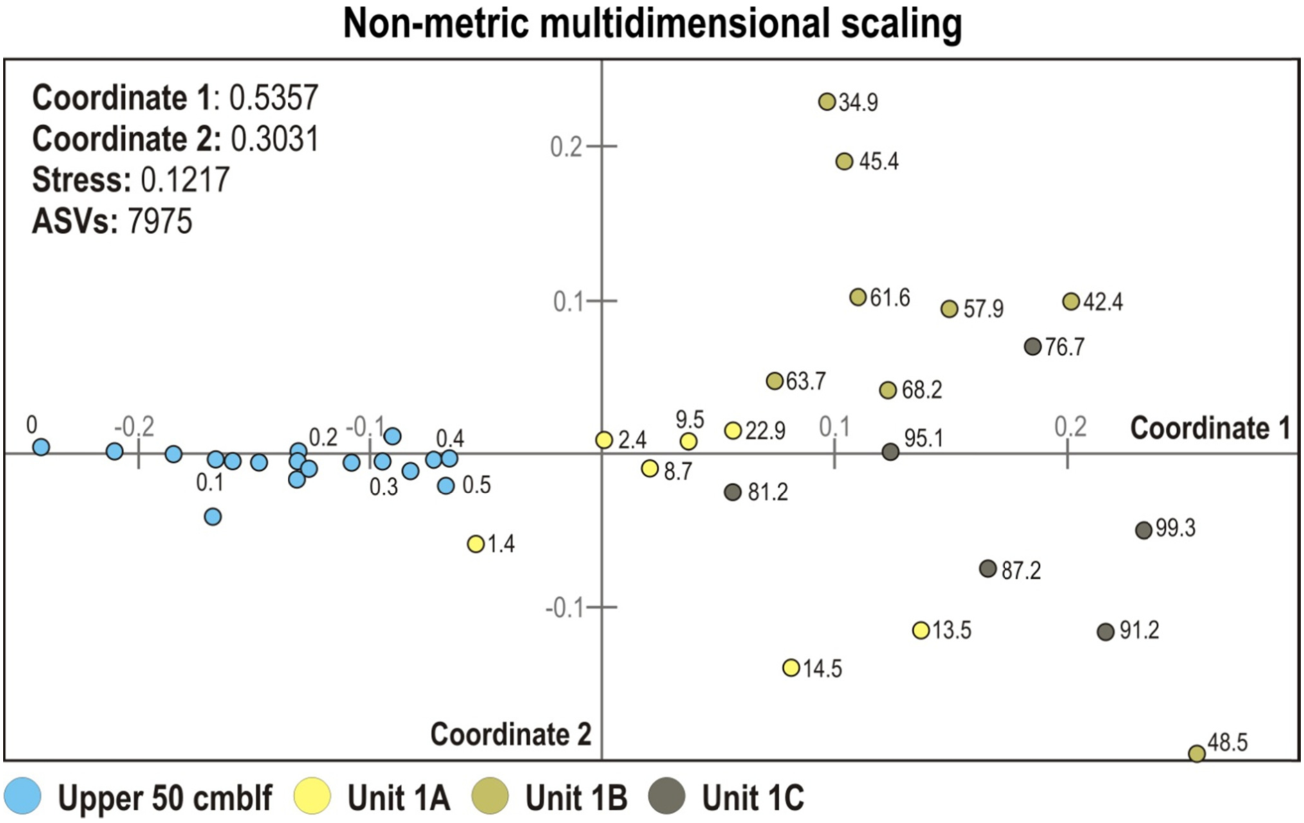

Supplementary Figure S7. Non-metric multidimensional scaling (NMDS) based on all 7975 ASVs recovered from 39 samples in Unit 1. In the NMDS plot, samples are distributed according to substrate depletion with sediment depth (coordinate 1) and lithological variations across stratigraphic units (coordinate 2). The numbers next to the samples (dots) correspond to sediment depths [mblf].

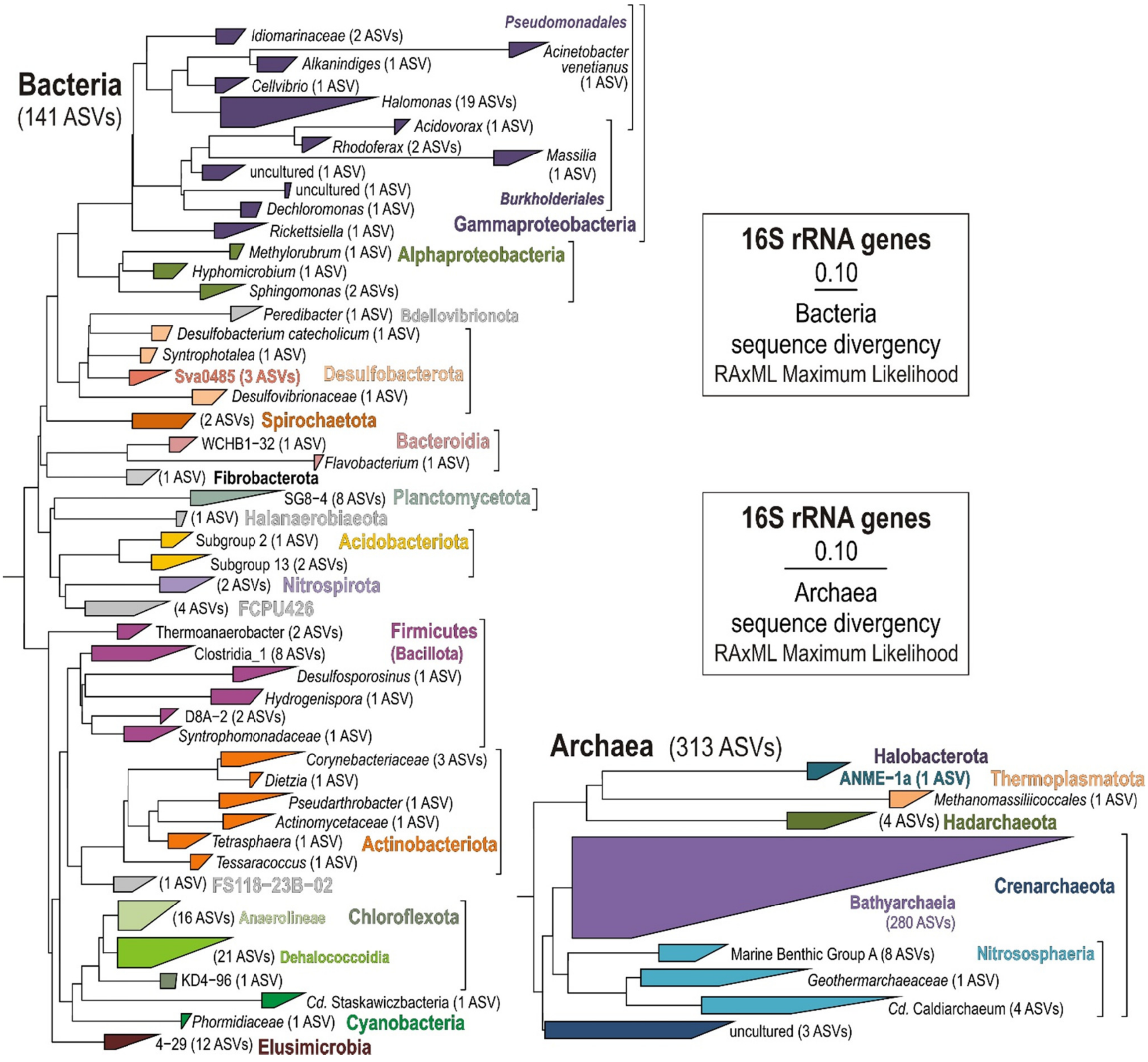

Supplementary Figure S8. Phylogenetic tree of 16S rRNA genes (V4 hypervariable region) for the amplicons sequenced in this study common to all three stratigraphic units. The phylogenetic trees for Bacteria (**left**) and Archaea (**right**) include the amplicon variant sequences (ASVs) common to all three stratigraphic Unit 1a, Unit 1b and Unit 1c (i.e. 454 ASVs). Boldface types signify cultivated species and sequence accession numbers to the rRNA SSU database release 138. Partial 16S rRNA gene amplicons (500 bps) were inserted in the SILVA NR99 reference tree on ARB, applying the ARB Parsimony algorithm with the bacterial and archaeal filters.

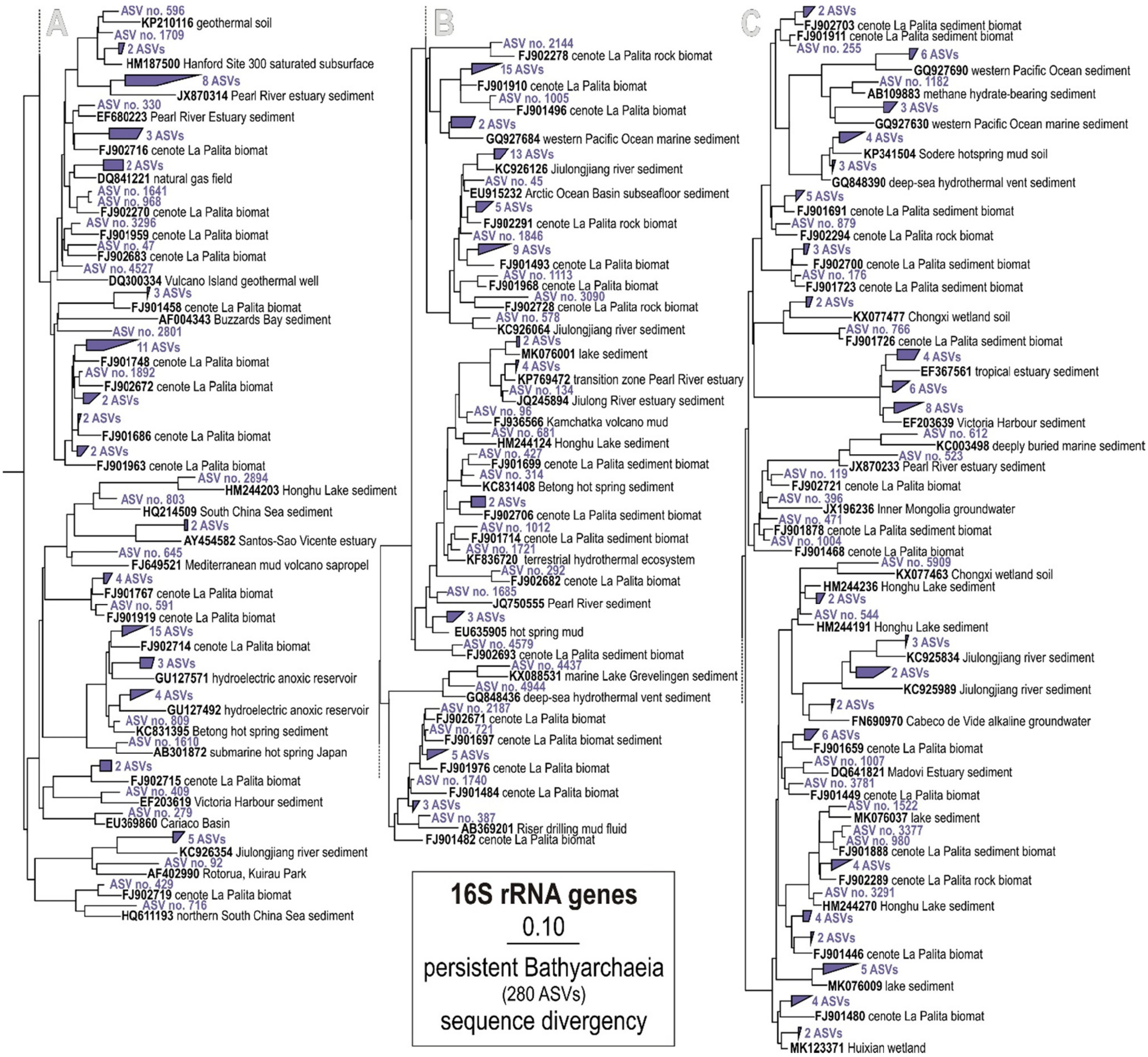

Supplementary Figure S9. Phylogenetic tree of 16S rRNA genes (V4 hypervariable region) for persistent Bathyarchaeia. The phylogenetic tree includes the amplicon variant sequences (ASVs) assigned to Bathyarchaeia that are common to all three stratigraphic Unit 1a, Unit 1b and Unit 1c (i.e. 280 ASVs). Boldface types signify cultivated species and sequence accession numbers to the rRNA SSU database release 138. Partial 16S rRNA gene amplicons (500 bps) were inserted in the SILVA NR99 reference tree on ARB, applying the ARB Parsimony algorithm with the bacterial and archaeal filters.

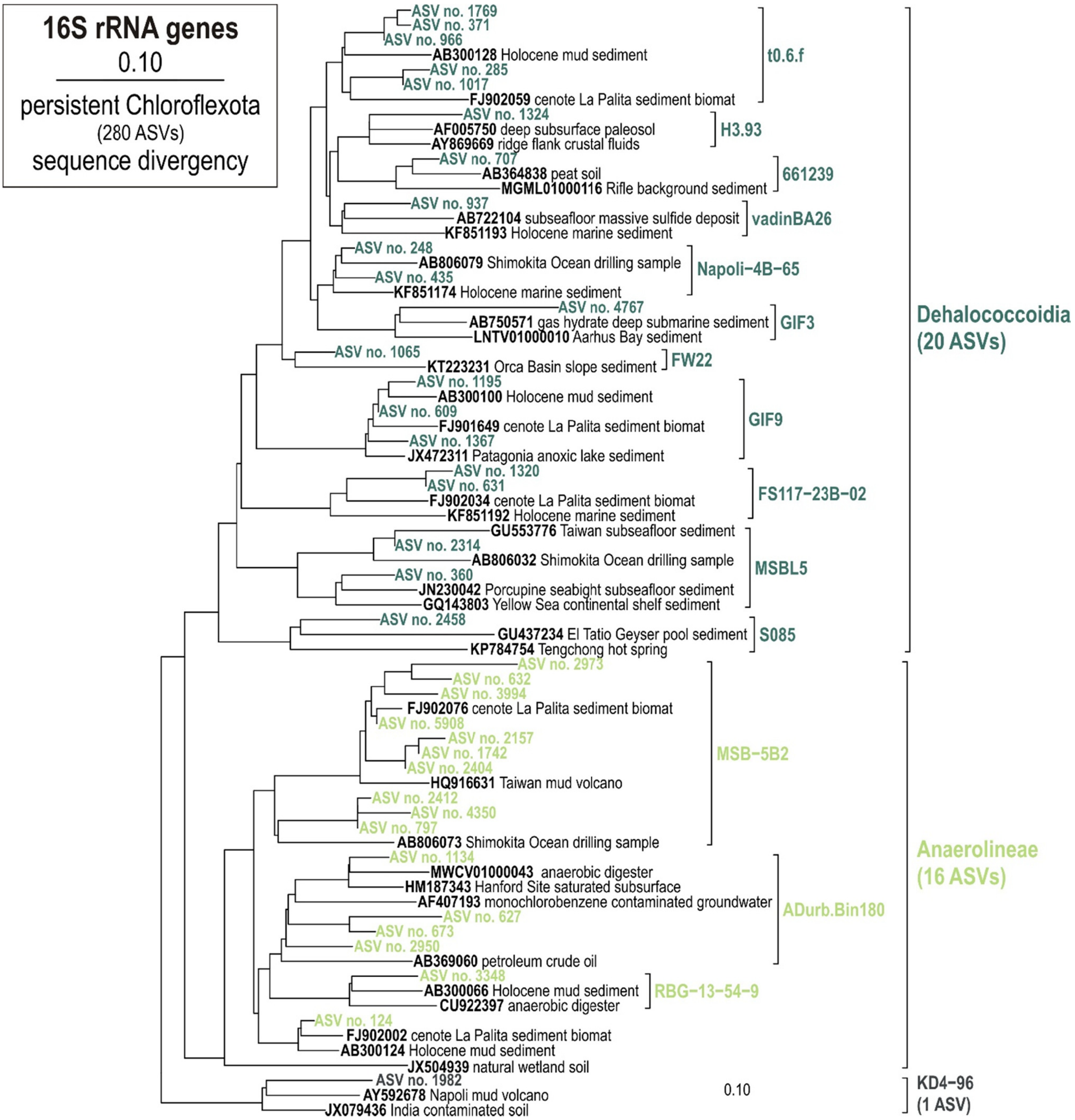

Supplementary Figure S10. Phylogenetic tree of 16S rRNA genes (V4 hypervariable region) for persistent Chloroflexota. The phylogenetic tree includes the amplicon variant sequences (ASVs) assigned to Chloroflexota that are common to all three stratigraphic Unit 1a, Unit 1b and Unit 1c (i.e. 37ASVs). Boldface types signify cultivated species and sequence accession numbers to the rRNA SSU database release 138. Partial 16S rRNA gene amplicons (500 bps) were inserted in the SILVA NR99 reference tree on ARB, applying the ARB Parsimony algorithm with the bacterial and archaeal filters.

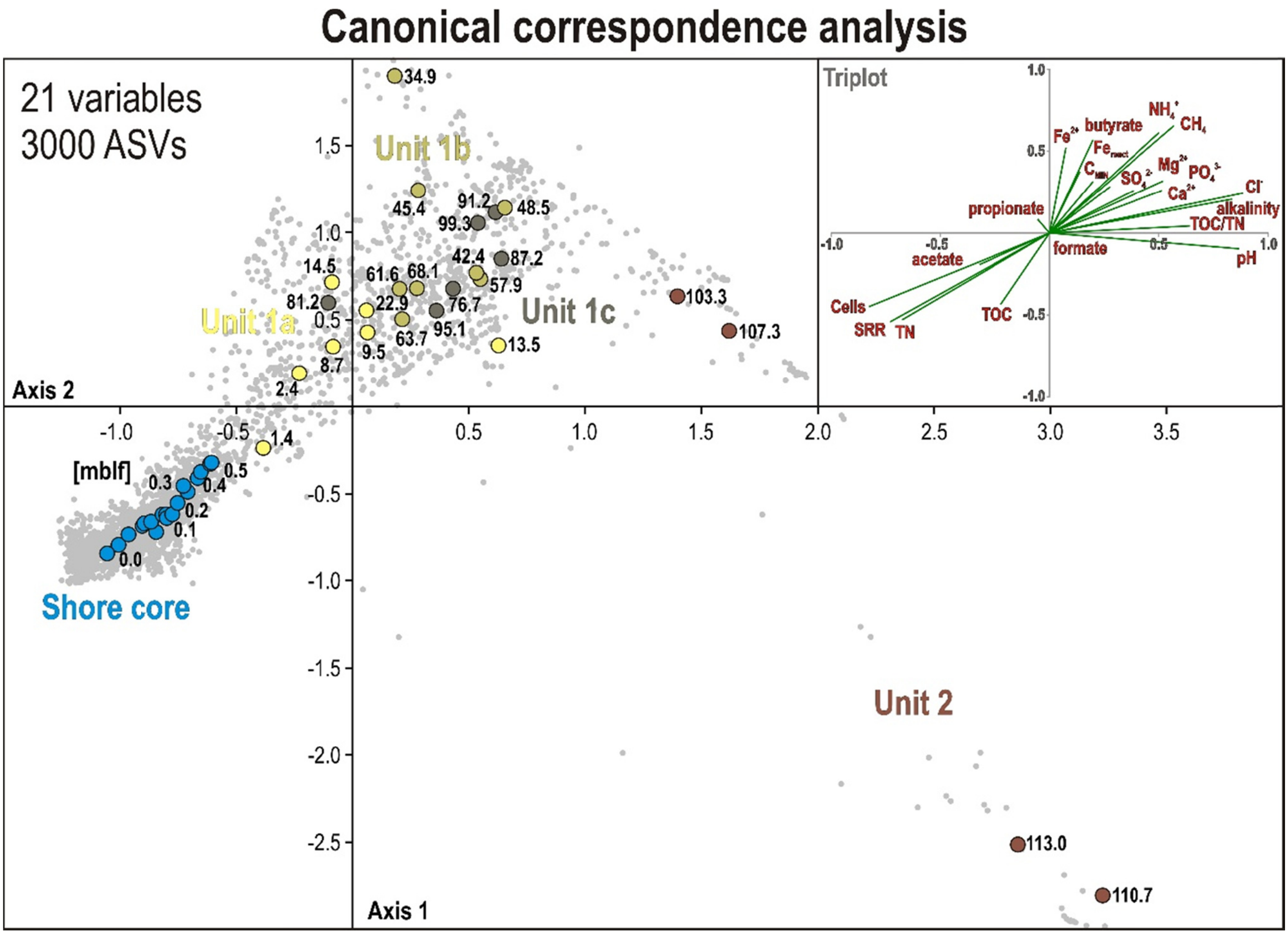

Supplementary Figure S11. Canonical correspondence analysis computed with samples originating from Unit 2. The CCA is based on 21 explanatory variables and the 3000 most abundant amplicon sequence variants (ASVs) for 43 samples including those from Unit 2. The distribution of samples (colored circles) and ASVs (grey dots) plots according to sediment depth. Explanatory variables (triplot) that significantly influence sample distribution with sediment depth are sulfate reduction rates (SRR), cell counts, pore water Fe^{2+}, NH_4^+, CH_4 concentrations, reactive iron (Fe_{react}) and mineral carbon content (C_{MIN}).

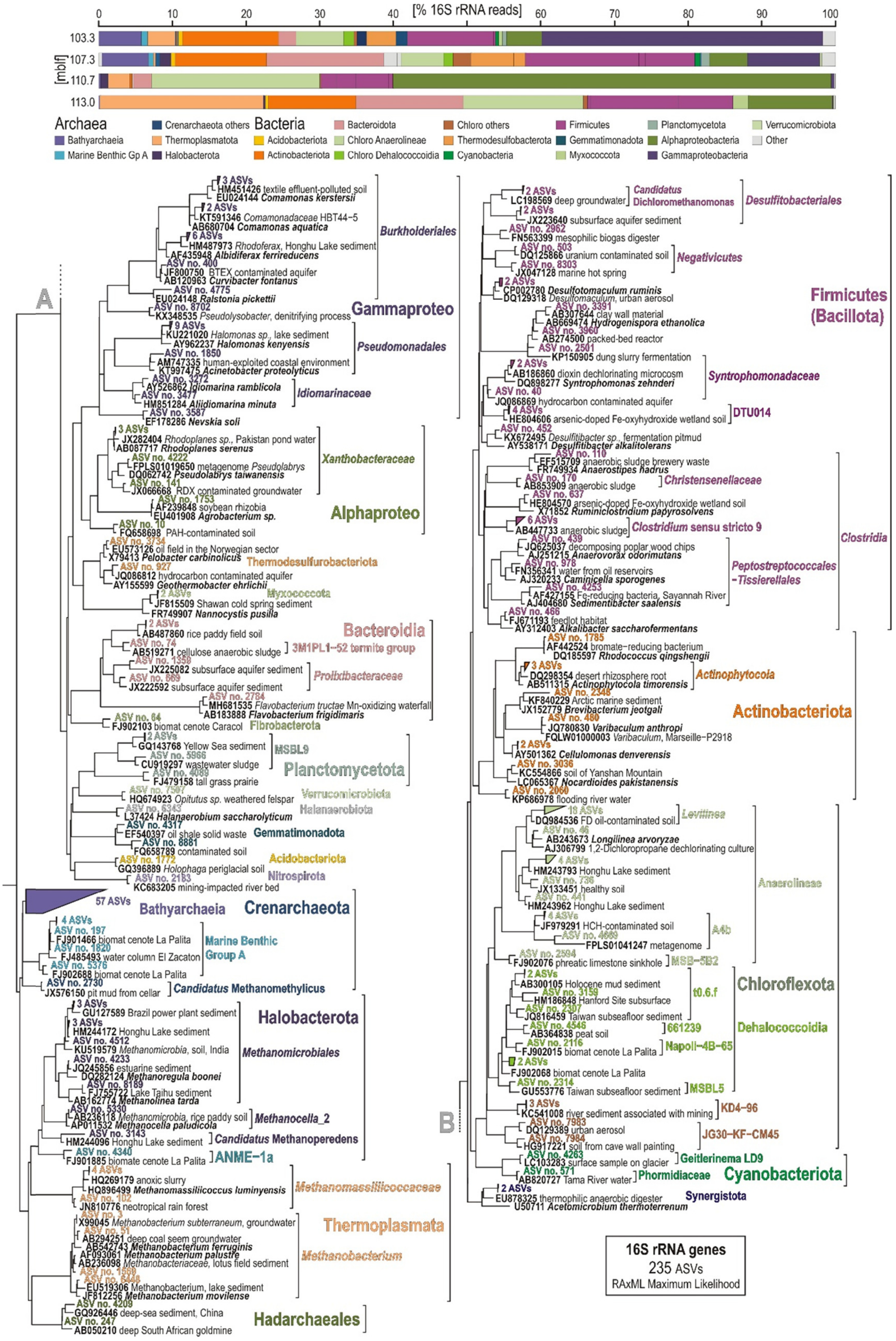

Supplementary Figure S12. Bar charts and phylogenetic tree of 16S rRNA genes (V4 hypervariable region) for the amplicons sequenced in this study originating from Unit 2. Relative abundance of 16S rRNA amplicon reads at the phylum/class level (**top**) and phylogenetic tree (**bottom**) including the amplicon variant sequences (ASVs) specifically identified in Unit 2 (i.e. 235 ASVs). Boldface types signify cultivated species and sequence accession numbers to the rRNA SSU database release 138. Partial 16S rRNA gene amplicons (500 bps) were inserted in the SILVA NR99 reference tree on ARB, applying the ARB Parsimony algorithm with the bacterial and archaeal filters.

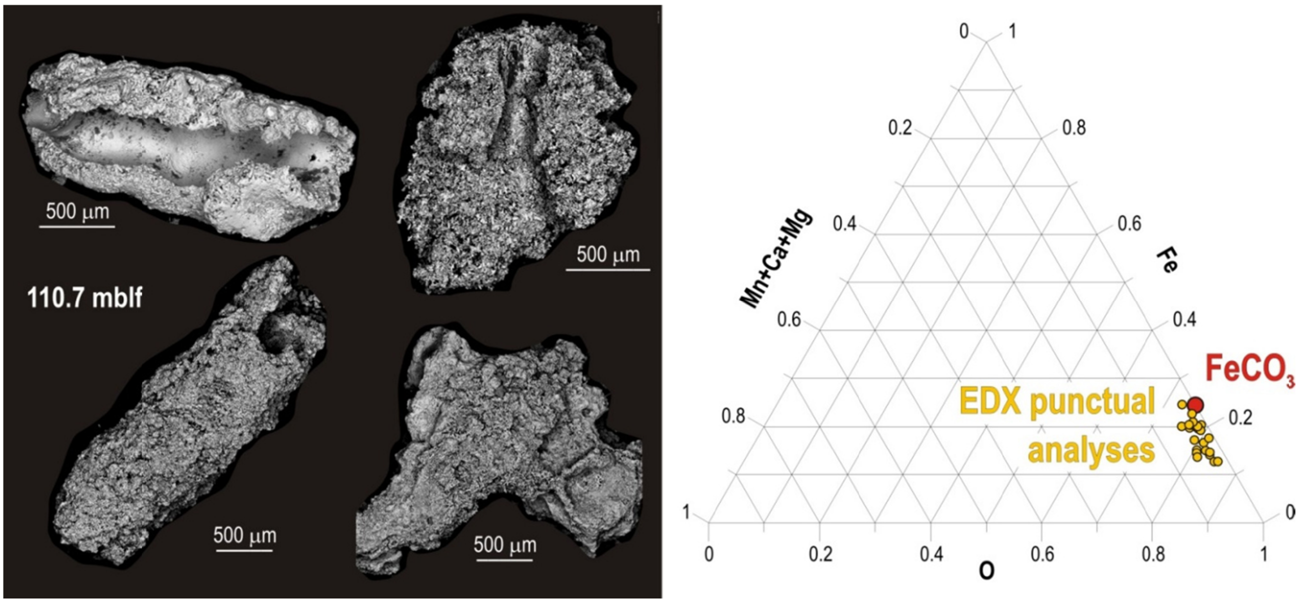

Supplementary Figure S13. Scanning-electron microscope imaging and EDX punctual analysis of siderite concretions from Unit 2. The scanning-electron microscope (SEM) images in back-scattered electron (BSE) mode show casts of plant residues incrusted by siderite (**left**), with EDX punctual analyses (**right**) confirming that siderite precipitated around rootlets as a coating mineral. Samples were collected from the top sequence of Unit 2 below the basal peat layer.
